# Population pharmacokinetics and pulmonary modeling of eravacycline and the determination of microbiological breakpoint and cutoff of PK/PD

**DOI:** 10.1128/aac.01065-24

**Published:** 2025-01-29

**Authors:** Xi-wei Ji, Wen Yao Mak, Feng Xue, Wen-yu Yang, Isabelle Hui-San Kuan, Xiao-qiang Xiang, Yun Li, Xiao Zhu

**Affiliations:** 1 Institute of Clinical Pharmacology, Peking University First Hospital 26447 , Beijing, China; 2 Department of Clinical Pharmacy and Pharmacy Administration, School of Pharmacy, Fudan University 598252 , Shanghai, China; 3 Momentum Metrix, San Francisco, California, USA; Providence Portland Medical Center, Portland, Oregon, USA

**Keywords:** eravacycline, population pharmacokinetics, pulmonary distribution, PK/PD

## Abstract

Eravacycline is a broad-spectrum fluorocycline currently approved for complicated intra-abdominal infections (cIAIs). In lung-infection models, it is effective against methicillin-resistant *Staphylococcus aureus* (MRSA) and tetracycline-resistant MRSA. As such, we aimed to develop a population pharmacokinetic/pharmacodynamic (PK/PD) model to evaluate eravacycline’s pulmonary distribution and kinetics. Data were extracted from a Phase I study (NCT01989949) which assessed the bronchopulmonary disposition of intravenous eravacycline to construct the population PK model that could adequately describe the drug’s pulmonary kinetics. Eravacycline lung PK was best described by a three-compartment model with allometric scaling, with the epithelial lining fluid (ELF) component parameterized as the ELF distribution ratio ($Ratio=\frac{C_{free,ELF}}{C_{free,\;central}}$, unbound concentration in ELF over central compartment). The estimated ELF distribution ratio was 8.26 (95% confidence interval = 6.8–9.8). Besides allometrically scaled weight, no other significant covariate was found. MIC_90_ was 0.5 mg/L (*Escherichia coli*), 2 mg/L (*Klebsiella pneumoniae*), 0.5 mg/L (*Acinetobacter baumannii*), and 0.12 mg/L (*S. aureus*). At the approved cIAI dosage or higher (1 mg/kg or 1.5 mg/kg q12h), a PK/PD cutoff value of 2 mg/L was appropriate for *E. coli*, while a lower value of 1 mg/L was selected for *K. pneumoniae*, *A. baumannii*, and *S. aureus*. For lower doses, the cutoff value was reduced to 0.5 mg/L for *K. pneumoniae*, *A. baumannii*, and *S. aureus*. The study showed eravacycline was widely distributed into the lungs with promising antibacterial efficacy, thus justifying further investigations into its uses for pulmonary infections.

## INTRODUCTION

Eravacycline is a fully synthetic fluorocycline with an excellent antimicrobial potency against many multidrug-resistant (MDR) gram-negative and gram-positive aerobic and anaerobic pathogens (1–5). It had recently received approval from the U.S. Food and Drug Administration (FDA) and China National Medical Products Administration for complicated intra-abdominal infections (cIAIs) (6, 7). When tested in healthy volunteers, intravenous eravacycline was extensively distributed into the epithelial lining fluid (ELF) and alveolar macrophages (8). These results supported further research to identify suitable susceptibility breakpoints of eravacycline for patients with respiratory infections.

Susceptibility breakpoints play an important role in antimicrobial agent selection. The Clinical and Laboratory Standard Institute (CLSI) recommended different categories of data to be used in establishing a susceptibility breakpoint, including pharmacokinetic/pharmacodynamic (PK/PD) cutoff value (9). The PK/PD cutoff value is defined as the minimum inhibitory concentration (MIC) that corresponds to a 90% probability of target attainment (PTA) based on a predetermined PK/PD index. For eravacycline, the index is the ratio of the area under the concentration-time curve to MIC (*f*AUC/MIC) (6, 10–12).

To assess eravacycline efficacy profile in pulmonary infections, we sought to integrate previously collected *in vitro*, *in vivo* (IVIV), and clinical data into a quantitative framework through modeling and simulation (M&S), and to predict eravacycline’s effect against certain clinically important bacteria of pulmonary infections. Specifically, we aimed to (i) develop a population PK model to describe the kinetics of free eravacycline in plasma and ELF, (ii) assess the PTA of five dosing regimens based on predetermined PK/PD target values, and (iii) determine the PK/PD cutoff values against four clinical bacteria. The study should provide important evidence to support further consideration of eravacycline for pulmonary infections.

## RESULTS

### Eravacycline pulmonary distribution population PK model

**Fig 1:**
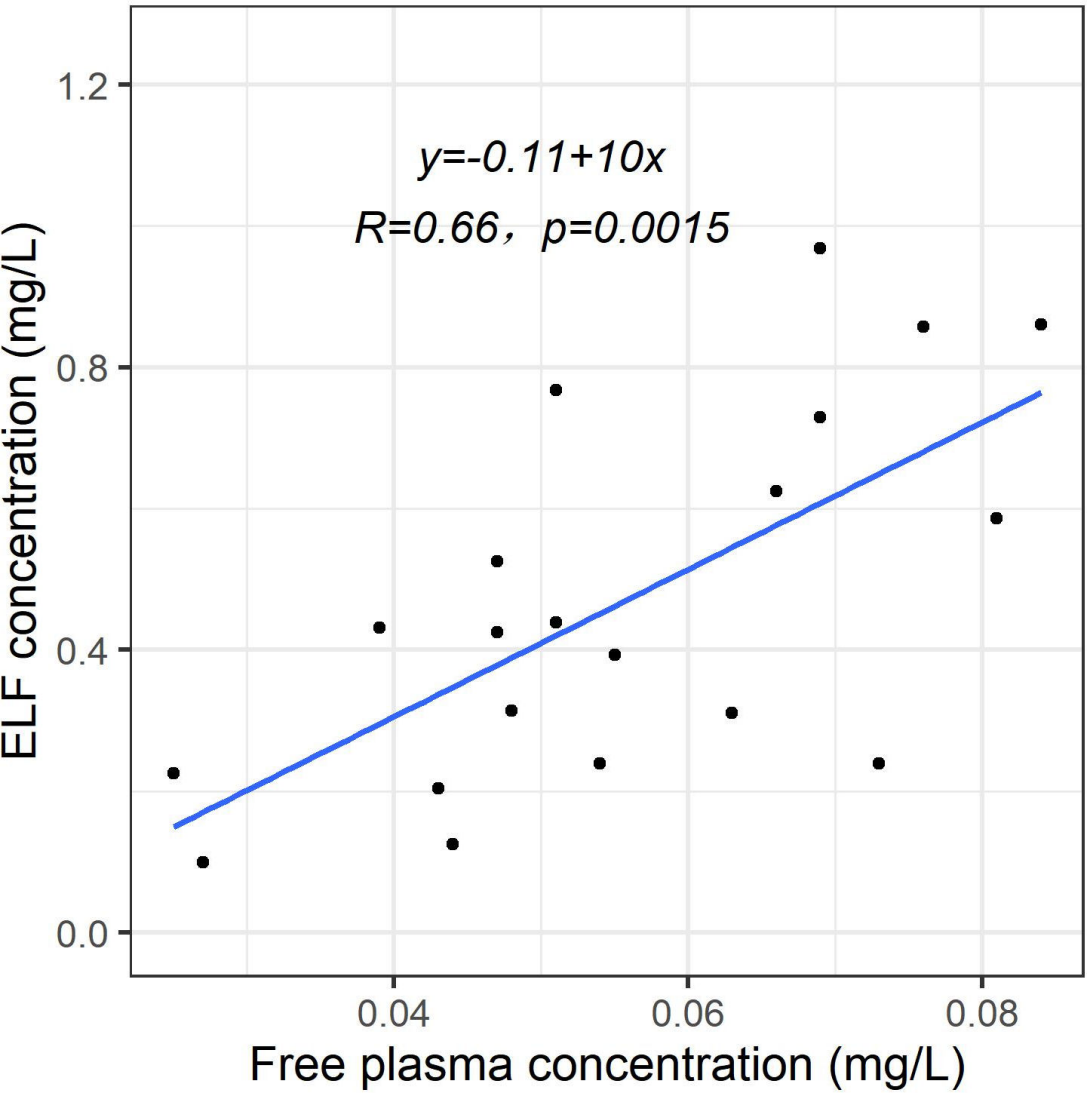
Correlation between free plasma eravacycline and ELF concentration.

**Fig 2:**
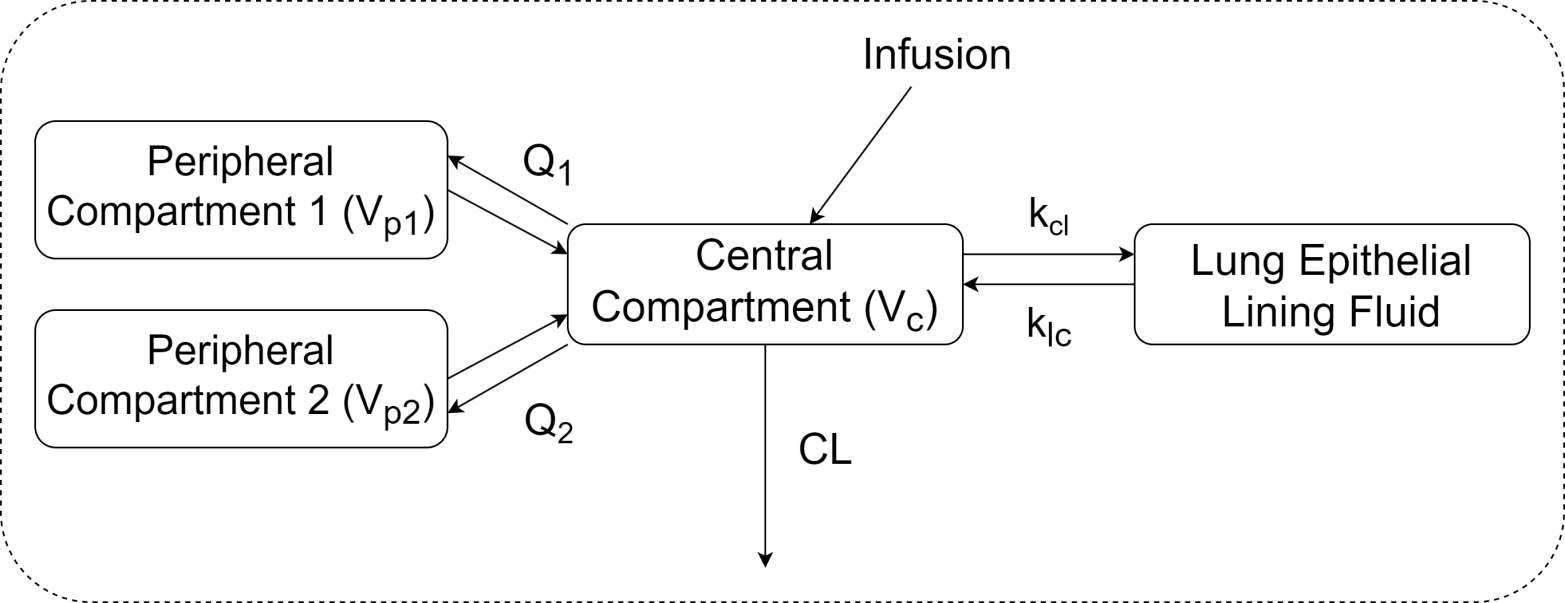
Schematic diagram of the final three-compartment model with allometric scaling and ELF distribution ratio. Abbreviations: *V*_P1_, volume of first peripheral compartment; *V*_P2_, volume of second peripheral compartment; *V*_c_, volume of central compartment; CL, systemic clearance; *k*_cl_ and *k*_lc_, rate constant between the central and ELF compartment; *Q*_1_, intercompartmental clearance with first peripheral compartment; *Q*_2_, intercompartmental clearance with second peripheral compartment.

The population pharmacokinetic of eravacycline in the lungs was best described by a three-compartment model as it produced the lowest value of Akaike information criterion, AIC (ΔAIC = −234 and −2.9 compared to one- and two-compartment model, respectively), and had a reduction in objective function value of 8.9 (*P* < 0.05, associated with two degrees of freedom) when compared to the two-compartment model. In addition, shrinkage for the central volume of distribution parameters was less for the three-compartment model (19% vs 36%), and the precision of the clearance parameter was marginally better (8% vs 10%). A total of 20 bronchoalveolar lavage (BAL) samples were collected (one BAL sample per subject), of which drug concentration could be determined in 15 samples and 5 below-quantification-limit (BQL) samples. The ELF component was parameterized as the ELF distribution ratio, $Ratio=\frac{C_{free,ELF}}{C_{free,\;central}}$ due to imprecise estimation of the intercompartmental clearance ($k_{cl}$ and $k_{lc}$) from sparse BAL sampling. Free eravacycline concentration in the plasma and ELF were found to be highly correlated (*R* = 0.66), thus providing a statistical basis for the simplification of the ELF component (see Fig. 1). Eravacycline concentration in ELF also demonstrated a reducing trend similar to that in the plasma concentration (see Fig. S3). The inclusion of *body weight* into the model via allometric scaling significantly improved model fit (ΔAIC=−13.8). No other covariate was included in the final model. The schematic of the final model is shown in Fig. 2. The key model development steps are summarized in the supplemental material (see Tables S1 and S2). All parameters were estimated with good precision (see Table 1).

**TABLE 1:** Parameter estimates of the final model and bootstrap results^*a*^ ^*b*^

Parameter (unit)	Estimates	RSE [SHR]	Bootstrap median (95% CI)
Fixed-effect parameters			
CL (L/h)	16.3	5%	16.2 (15.0–17.4)
*V*_c_ (L)	3.88	25%	3.5 (1.8–7.2)
*Q*_1_ (L/h)	33.9	11%	32.7 (24.7–47.7)
*V*_P1_ (L)	39	20%	37.6 (29.2–54.5)
*Q*_2_ (L/h)	23.5	16%	23.3 (19.2–29.2)
*V*_P2_ (L)	122	15%	121 (109.9–134.9)
Ratio	8.26	12%	8.3 (6.8–9.8)
Interindividual variability			
CL	13.6%	69% [0%]	13.2% (9.7–16.5%)
*V*_c_	116%	49% [19%]	108.9% (63.8–146.7%)
*V*_P1_	55%	56% [8%]	54.4% (32.3–75.5%)
Residual variability (serum concentration)			
Proportional error	7.04%	21% [22%]	6.82% (4.23–9.24%)
Additive error	8.73	48% [22%]	9.02 (0.19–16.1)
Residual variability (BAL concentration)			
Proportional error	42.3%	28% [0%]	41.4% (29.0–52.5%)

^*a*^ $CL=16.3\;\times {\left(\frac{WT}{81.4}\right)}^{0.75}L/h;V_{c}=3.88\;\times \left(\frac{WT}{81.4}\right)L;Q_{1}=33.9\;\times {\left(\frac{WT}{81.4}\right)}^{0.75}L/h;V_{p1}=39\;\left(\frac{WT}{81.4}\right)\times L;\;Q_{2}=23.5\;\times {\left(\frac{WT}{81.4}\right)}^{0.75}L/h;\;V_{p2}=\;122\;\times \left(\frac{WT}{81.4}\right)L$

^*b*^ Abbreviations: BAL, bronchoalveolar lavage; CI, confidence interval; CL, clearance; Q1 and Q2, intercompartmental clearance of the first and second peripheral compartment; Ratio, the ELF-to-plasma concentration ratio; RSE, relative standard error; SHR, shrinkage; Vc, volume of distribution of central compartment; Vp1 and Vp2, volume of distribution of the first and second peripheral compartment; WT, weight .

**Fig 3:**
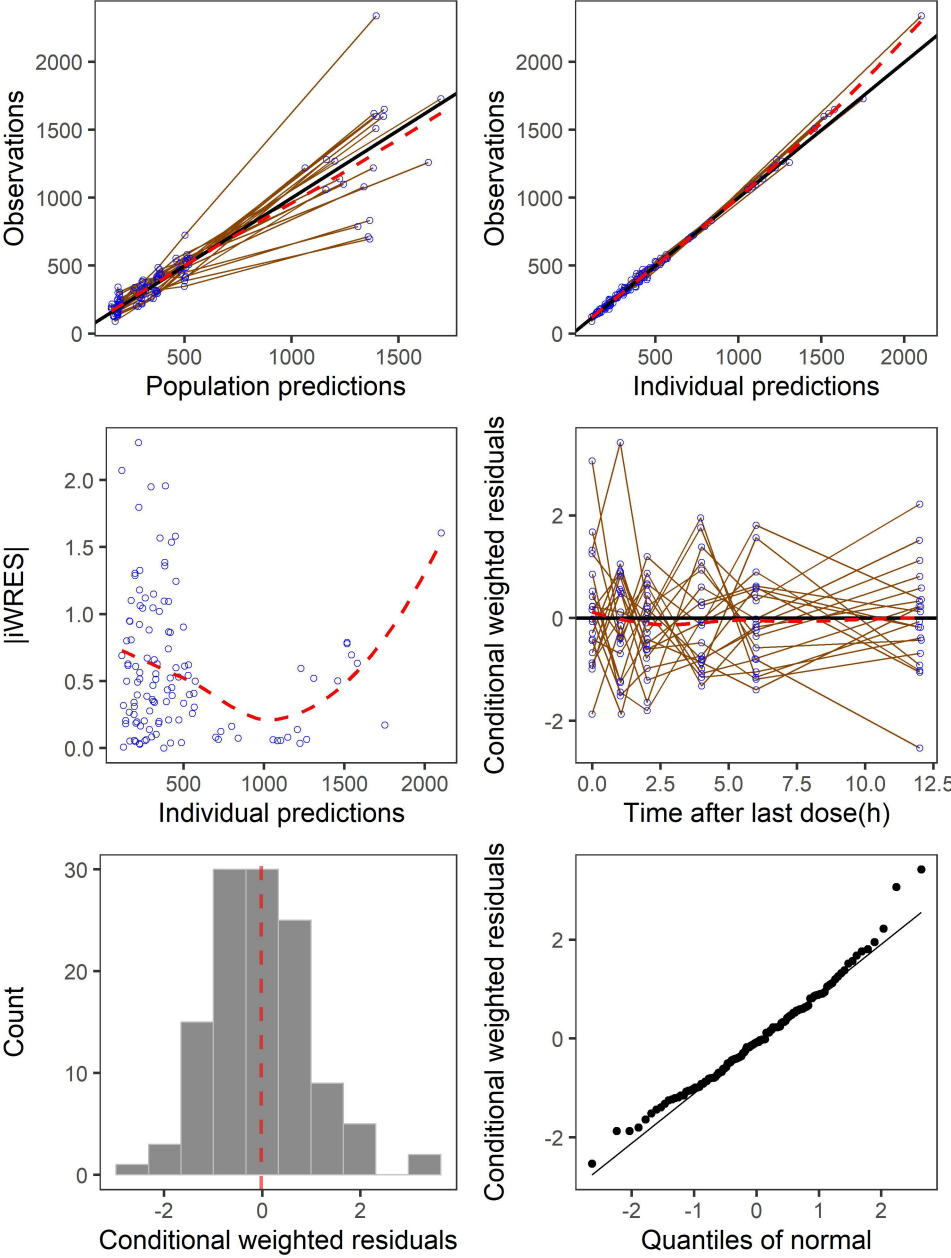
Goodness-of-fit plots of eravacycline final model using plasma data. iCWRES, individual conditional weighted residuals.

**Fig 4:**
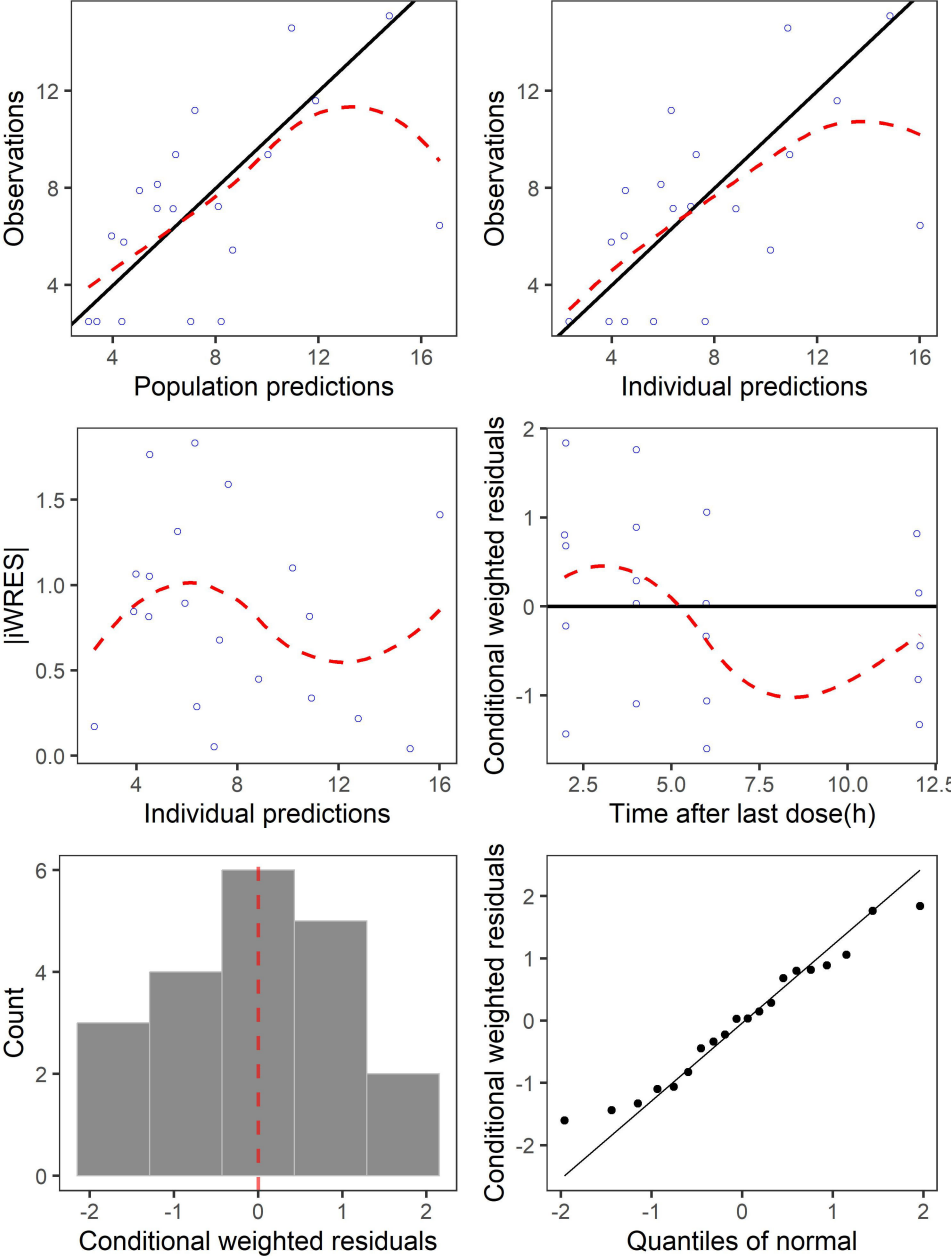
The goodness-of-fit plots of eravacycline final model using bronchoalveolar lavage (BAL) data. iCWRES, individual conditional weighted residuals.

**Fig 5:**
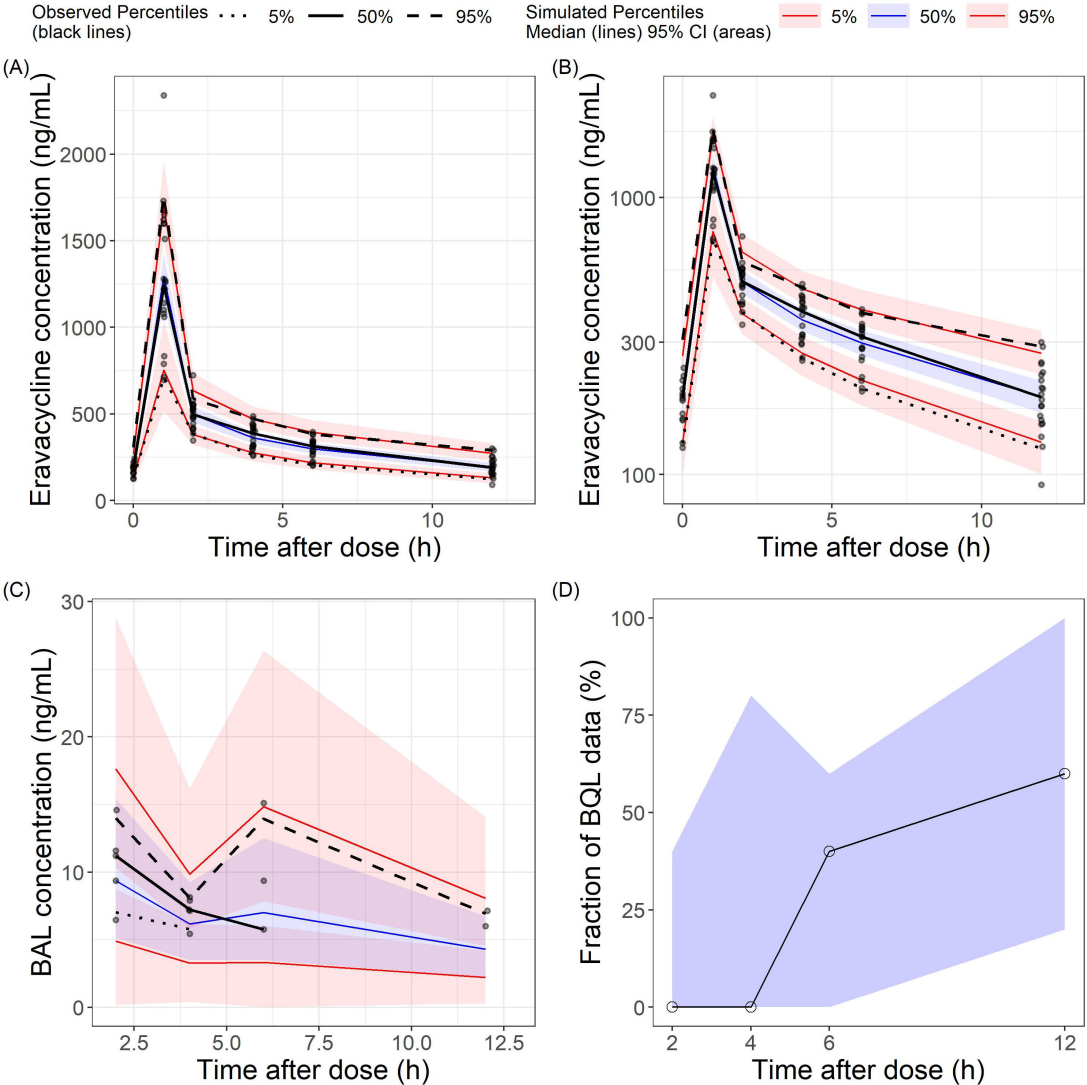
Visual predictive check (VPC) of the final model for (**A**) plasma eravacycline in arithmetic scale, (**B**) plasma eravacycline in logarithmic scale, and (**C**) bronchoalveolar lavage (BAL) eravacycline in arithmetic scale. (**D**) The fraction of below-quantitative-limit (BQL) values in the observed data set.

The goodness-of-fit (GoF) plots suggested the final model was a good fit for both the plasma and BAL eravacycline data (Fig. 3 and 4). Visual predictive check (VPC) results of eravacycline concentration in plasma and BAL showed that most of the observed values were contained within the 90% prediction intervals, thus indicating that the final model could predict the concentrations with acceptable accuracy (Fig. 5). Bootstrap results suggested that the estimated values of the final model parameters were close to the median and within the 95% CI from the non-parametric bootstrap.

### *In vitro* susceptibility toward eravacycline

The *in vitro* susceptibility of all four bacteria towards eravacycline is shown in Table 2. The MIC_90_ of eravacycline was 0.5, 2, 0.12, and 0.5 mg/L, respectively, against *Escherichia coli*, *Klebsiella pneumoniae*, *Staphylococcus aureus*, and *Acinetobacter baumannii*.

**TABLE 2:** *In vitro* antibacterial efficacy of eravacycline^*a*^

Type of bacteria	MIC_50_ (mg/L)	MIC_90_ (mg/L)	MIC distribution (mg/L)													Total isolates
			0.004	0.008	0.16	0.03	0.06	0.12	0.25	0.5	1	2	4	8	16	
*E. coli*	0.12	0.5	/	/	/	9	67	223	142	41	12	6	/	/	/	500
*K. pneumoniae*	0.5	2	/	/	/	1	8	66	157	165	44	38	14	6	1	500
*A. baumannii*	0.06	0.5	23	14	63	134	72	47	29	84	22	8	4	/	/	500
*S. aureus*	0.03	0.12	1	52	134	121	114	33	14	19	12	/	/	/	/	500

^*a*^ Abbreviations: MIC, minimum inhibitory concentration; MIC_50_ and MIC_90_, the MIC values that inhibit 50% and 90% of isolates, respectively. /, not tested.

### Determination of PTA and cumulative fraction of response (CFR)

**Fig 6:**
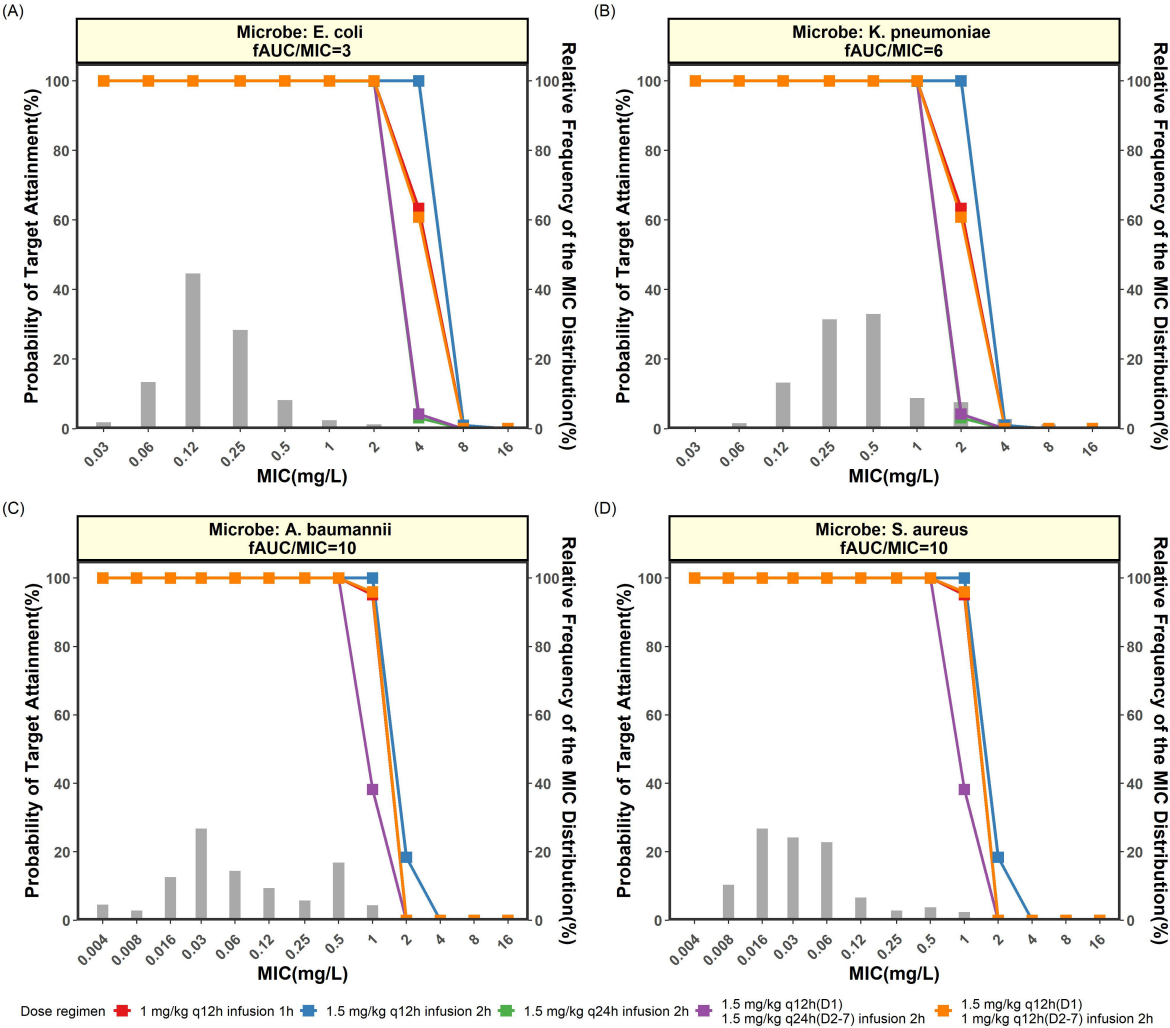
Probability of target attainment of eravacycline under different dosing regimens against (**A**) *E. coli* at *f*AUC/MIC = 3, (**B**) *K. pneumoniae* at *f*AUC/MIC = 6, (**C**) *A. baumannii* at *f*AUC/MIC = 10, and (**D**) *S. aureus* at *f*AUC/MIC = 10. *f*AUC/MIC, fraction of area-under-the-curve over minimum inhibitory concentration; MIC, minimum inhibitory concentration; PTA, probability of target attainment

The simulated efficacy of eravacycline against all four bacteria at respective PK/PD targets and MIC values are summarized in Fig. 6. The corresponding PTA values at each level of MIC and the results of simulated efficacy for all other predetermined PK/PD targets are included in the supplemental material (see Table S4; Fig. S4 to S6).

For *E. coli*, the MIC distribution of eravacycline showed that all MIC values were ≤2 mg/L. At *f*AUC/MIC = 3, all five dosing regimens achieved a PTA of 100% given the MIC range from 0.03 to 2 mg/L. When the MIC value was increased to 4 mg/L, only regimen 2 (1.5 mg/kg q12h given over 2 h) provided adequate bacteriostatic effect (△log(CFU/g) = 0) with PTA = 100%. The CFR against *E. coli* was more than 99% for all five dosing regimens.

Several PK/PD targets were tested for *K. pneumoniae*. At *f*AUC/MIC values of 10 and 8, all dosing regimens could achieve a PTA of 100% only when the MIC values were within 0.03–0.5 mg/L. When the PK/PD target was set at a lower value of 6, regimen 2 could achieve a PTA of 100% at higher MIC values of 2 mg/L. The CFR values against *K. pneumoniae* at *f*AUC/MIC = 6 were above 90% for regimens 1, 2, and 5.

For *A. baumannii*, at the predetermined target of *f*AUC/MIC = 10, all five dosing regimens achieved a PTA > 90% for MIC values that ranged from 0.004 to 0.5 mg/L. At higher MIC values, the low-dose regimens (3 and 4) failed to achieve the desirable PTA. The corresponding CFR values at *f*AUC/MIC = 10 were >90% for all regimens.

For *S. aureus*, when *f*AUC/MIC = 10, all five dosing regimens could achieve a PTA > 90% when the MIC values ranged from 0.004 to 0.5 mg/L. Similarly, low-dose regimens 3 and 4 failed to achieve PTA at higher MIC values. The corresponding CFR was >98% for all regimens.

### Determination of PK/PD cutoff values

At the currently approved dosage (regimen 1) and proposed higher dosages (regimens 2 and 5), the appropriate PK/PD cutoff value for *E. coli* was 2 mg/L, while a halved cutoff of 1 mg/L was deemed appropriate for *K. pneumoniae*, *A. baumannii*, and *S. aureus* (Table 3). For the proposed lower doses (regimens 3 and 4), the PK/PD cutoff value for *E. coli* remained at 2 mg/L but was quartered to 0.5 mg/L for *K. pneumoniae*, *A. baumannii*, and *S. aureus* (see Table S5). The corresponding CFR values are tabulated in Table 4, which showed that CFR was mostly above 90% for all four tested bacteria.

**TABLE 3:** Target values and PK/PD cutoff values of eravacycline against different tested bacterium

Bacteria	Target value (*f*AUC/MIC)	PK/PD cutoff values (mg/L)
*E. coli*	3	2
*K. pneumoniae*	6	1
*A. baumannii*	10	1
*S. aureus*	10	1

**TABLE 4:** Cumulative fraction of response values for each dosing regimens against the four bacteria

Bacteria	*f*AUC/ MIC	Regimen 1	Regimen 2	Regimen 3	Regimen 4	Regimen 5
*E. coli*	3	100%	100%	100%	100%	100%
*K. pneumoniae*	6	93.0%	95.8%	88.4%	88.5%	92.8%
*A. baumannii*	10	97.4%	97.9%	94.9%	94.9%	97.4%
*S. aureus*	10	99.9%	100%	98.5%	98.5%	99.9%

## DISCUSSION

The pulmonary distribution population PK model suggested that eravacycline was widely distributed in the lungs after intravenous administration. To our knowledge, this is the first attempt to model eravacycline’s kinetics in the human lungs. ELF distribution was increased eightfold and this showed a high penetration of eravacycline into the lungs. In addition, weight was found to positively correlate with clearance. The correlation was not linear but less than proportionality (weight raised to a power of 0.62). This may explain why AUC was still correlated with weight after weight-based dosing (the increase in AUC was approximately proportional to weight raised to the power of 0.36, see Fig. S1).

Although published values of eravacycline clearance fluctuate between studies (8, 13–15) few substantial changes were observed when normalized to the subject’s weight (reported CL values ranged from 0.16 to 0.23 L/h/kg; see oTable S3 ). Connors et al. estimated eravacycline CL as 17.8 L/h but stated that the *C*_max_ had an approximately 30% reduction compared to Sutcliff et al. (data not available) (8, 16). This observation was attributed to the increased volume of distribution (ca. 35% greater) as some volunteers had body mass index (BMI) >33.0 kg/m^2^. Nonetheless, Asempa et al. demonstrated eravacycline had consistent therapeutic utility with a high clinical cure rate (82–94%) across different BMI categories (17).

Drug concentrations in the ELF are considered appropriate proxies for antibiotic concentration at pulmonary target sites (18) to gauge antibiotic activities. Ceftriaxone—a widely used beta-lactam antibiotic for community-acquired pneumonia (CAP)—has a 12.2 ± 5.2 times higher concentration in ELF than in plasma (19). In this study, eravacycline concentration in the ELF was 8.3 times higher than plasma, which approximated the value observed in ceftriaxone for CAP. Compared to the congener tigecycline that had a significantly lower ELF concentration (penetration ratio ranged from 1.15 to 3.3, as demonstrated in Rubino et al. and Kiem and Schentag) (18, 20), eravacycline appeared to have a better penetration into lung tissues. Moreover, although eravacycline is widely distributed in the body (21), it has comparatively lower volume of distribution against tigecycline (4 L/kg vs. 6–9 L/kg of tigecycline), which corresponds to higher plasma and lung exposure (9.12 vs 3.70 µg·h/mL and 9.18 vs 6.32  µg·h/mL, respectively) (22, 23). This eravacycline PK profile may offer advantages in treating pulmonary infections.

On average, all five dosing regimens demonstrated good efficacy against the tested bacteria with CFR values of more than 90%. The corresponding PK/PD cutoff values were higher for *E. coli* compared to *K. pneumoniae*, *A. baumannii*, and *S. aureus*, reflecting the relative susceptivity between different bacteria toward eravacycline. These results provided reasonable scientific support to consider further investigations into eravacycline’s application in pneumonia or other pulmonary infections.

The selection of PK/PD target has paramount importance in the development of an antimicrobial agent. *In vivo* studies indicated that the *f*AUC/MIC targets obtained from neutropenic or immunocompetent models were substantially different. Using neutropenic murine thigh model, Zhao et al. demonstrated that the median *f*AUC/MIC target value of eravacycline against *E. coli* was 32.59 (11), but the median target value in immunocompetent models was significantly lower at 8.5 (10). This phenomenon was observed in tigecycline against different isolates of *E. coli* and *K. pneumoniae* as well where the presence of a competent immune system markedly reduced the exposure required to achieve bactericidal effects (24). Considering the MIC breakpoints for *E. coli* as published by the European Committee for Antimicrobial Susceptibility Testing (EUCAST) and the U.S. FDA, it can be inferred that the values predominantly reflect the outcomes from PK/PD targets acquired using immunocompetent *in vivo* models. In addition, eravacycline has been evaluated in four clinical studies for cIAI, including three randomized, double-blind, active-controlled, international multicenter trials (TP-434-P2-cIAI-1, TP-434-008, and TP-434-025) (1–3), and one Phase III, randomized, double-blind, active-controlled bridging study in China (TP-434-EM-003, unpublished results). These studies suggested that eravacycline was effective both microbiologically and clinically against infections caused by Enterobacterales with MIC values of 1 or 2 mg/L. In particular, when tested against 34 isolates of Enterobacterales with MIC of 1 mg/L, the reported microbiological and clinical efficacy was 88.2%; when MIC was 2 mg/L (*n* = 11), the efficacy was reported 81.8%. Against *A. baumannii* with MIC values of ≤1 mg/L, the efficacy was 100% (1–3). These clinical results supported the PK/PD target values used in this study.

Thus far, CLSI has not published the clinical breakpoints of eravacycline, and only the breakpoints of eravacycline against *E. coli* published by the U.S. FDA (25) and EUCAST (26) are consistent. Notably, these breakpoint values are based on plasma concentration data and may not extrapolate well for pulmonary infections. In turn, our study provided preliminary information to aid further investigation into eravacycline’s pulmonary PK/PD characteristics. Monte Carlo simulation shows that in healthy adults with normal renal function, 1 h infusion of 1 mg/kg q12h for 7 days, 2 h infusion of 1.5 mg/kg q12h for 7 days, and 2 h infusion of 1.5 mg/kg q12h on the first day, followed by 1 mg/kg q12h on second day onwards were adequate for MIC ≤2 mg/L (*E. coli*) and MIC ≤1 mg/L (*K. pneumoniae*, *A. baumannii*, and *S. aureus*), respectively. The result also suggested that the currently approved cIAI dosage is sufficient to cover MIC of 2 or 1 mg/L for the tested bacteria without needing the higher dosages. For the five dosing regimens of eravacycline in this study, the approaches of shortening the dosing interval, prolonging infusion time, and increasing dosage only exerted a limited impact on improving the PTA.

It is worth noting that the PK/PD cutoff values proposed in this study were higher than published clinical breakpoint values under the approved dosage of eravacycline (26, 27). This could be due to several reasons. First, the much higher lung concentrations (i.e., drug exposure) led to better effects from eravacycline that exhibited concentration-dependent killing. Second, the PK/PD target values used may differ for combinations of target organs and bacteria. It is not uncommon to observe large variations in target values for different diseases. Leng et al. reported that the target values of tigecycline ranged from 0.9 for bone and joint infection by susceptible *S. aureus*, *K. pneumoniae* or *E. coli*, to 100 for hospital-acquired infection caused by MDR *A. baumannii* or carbapenem-resistant *K. pneumoniae* (28). We have elected to test a wide range of target values in this study and select the maximum in determining the PK/PD cutoff values, recognizing the current lack of conclusive animal or *in vitro* PK/PD data on eravacycline against the tested bacteria (except *E. coli*). Discrepancies between PK/PD and clinical breakpoints were common for many antibiotics (29). For example, Barrasa et al. found that the PK/PD breakpoints of tigecycline and cefiderocol were higher than the ECOFF and clinical breakpoints published by CLSI. In these cases, exposure to antibiotics could be considered sufficient for isolates that had higher MIC values (resistant strain). The authors demonstrated that while PK/PD models are valuable in dosing optimization, variabilities in PK/PD targets and MIC distributions complicate direct clinical application. This is supported by a recent study by Hsueh et al. that re-estimated the susceptibility of pneumonia-causing meropenem-resistant (MEM-R) *A. baumannii* isolates against important antibiotics (30). They suggested higher doses of tigecycline (100 mg q12h IV) and minocycline (200 mg q12h IV) were necessary to effective treatment. The suggestion to double the routine IV maintenance dosages of tigecycline and minocycline was corroborated by Jean et al. (31).

M&S work in this study predicted promising efficacies for pulmonary infections albeit uncertainties in extrapolating findings into clinical effectiveness. Similar to tigecycline, we expected some degrees of difference between susceptibility breakpoints and PK/PD breakpoints when a standard eravacycline dose is used, particularly for drug-resistant strains. The important insights reported by Barrasa et al., Hsueh et al., and Jean et al. reinforce the need to reconsider susceptibility breakpoints for eravacycline using clinical data. Moreover, favorable outcomes based on eravacycline 1 mg/kg q12h combination therapy reported by Jackson et al. for carbapenem-resistant *A. baumannii* (CRAB) pneumonia in critically ill patients reinforced our findings (32). The study demonstrated adequate clinical success (71%) and microbiology resolution (71%), which supported the validity of PK/PD targets against *A. baumannii* at the standard dose. Further supportive evidence was recently published by the Chinese Expert Committee on Clinical Use of Antimicrobials and Evaluation of Antimicrobial Resistance. The committee recently published a report on the outcome of 3,369 off-label use cases of eravacycline, of which 62.5% were pulmonary infections, 7.1% combined pulmonary infection and bacteremia, and 5.0% combined pulmonary and abdominal infections (33). The standard dose of 1 mg/kg q12h was used in 94.6% of the cases, and the alternative 50 mg q12h dose was used in the remaining cases. The study found that 90.1% of patients achieved clinical resolution with eravacycline, with 90.7% observed microbiological resolution. Specifically, the clinical resolution rates against *A. baumannii* and *K. pneumoniae* were 86.0% and 86.7%, respectively (33). Another case series that reported 24 cases of ventilator-associated pneumonia due to CRAB suggested a lower clinical resolution rate at 71% (32). Nonetheless, extra caution must be exercised with off-label use of eravacycline, considering the susceptibility rates of eravacycline may differ between regions and use-cases. The use case of tigecycline for *A. baumannii* is an excellent reference where high doses should only be used for certain critical cases (30).

Our study has several limitations. The data used to develop the population PK model were derived from a modest cohort of 20 subjects, all below the age of 47 and were healthy. The restricted sample size and homogeneous demographic profile may not represent the broader patient population, and potentially overlooked critical covariates affecting PK dynamics. Second, the PK/PD target values established for *K. pneumoniae*, *A. baumannii*, and *S. aureus* were preliminary and derived from limited data. Despite the fact that we had adopted a cautious methodology by selecting higher target values, further empirical evidence is necessary to validate the proposed PK/PD cutoff values. Third, the four subjects who provided ELF samples with BQL concentrations constituted 20% of all ELF measurements. These BQL data were handled using the appropriate analysis methods as suggested in the 2022 FDA’s Guidance for Industry on Population Pharmacokinetics (34) and as recommended by modeling experts (35–38). In addition, BAL concentration at the 6th h demonstrated comparatively larger variations than other time points. Inspection of the data set suggested that the volunteers who contributed to these samples had a large variability in BMI (ranging from 23.3 to 31.6 kg/m^2^), which affected the dose administered (ranging from 71.7 to 94.3 mg). Nonetheless, plots of the dose-normalized ELF concentration and VPC of ELF concentration suggested the variability at this time point did not differ significantly from others, and the trend closely mimicked that of the plasma concentration (see Fig. S2 and S3). Finally, FDA highlighted the variability in PK/PD targets observed in animal models and uncertainties in plasma protein binding across species (13). These factors may introduce uncertainty when extrapolating breakpoint determination and underscore the need for caution when interpreting modeling results derived from preclinical data and applying them to clinical practice. Furthermore, the breakpoints applied to the PK/PD cutoffs originated from *in vitro* data as there was no human data on the use of eravacycline for pulmonary infections when this work was completed. It should be recognized that clinical breakpoints should be used whenever possible. With the most recent publication of the outcomes from eravacycline use under different clinical scenarios in China (33), it is expected that more real-world clinical data will become available. These data will provide a stronger foundation for validating PK/PD breakpoints and refining clinical breakpoints for eravacycline.

### Conclusion

This study demonstrated that eravacycline, when given via intravenous infusion, is distributed widely into the lungs. Monte Carlo simulations suggested effectiveness against four important pathogens associated with pulmonary infections. While reported variability in PK/PD targets observed in animal models and uncertainties in plasma protein binding hindered the establishment of a definitive breakpoint, the findings provide a robust overview of PK/PD thresholds under various dosing regimens. This work contributed to the integration of prior knowledge via M&S, which supports eravacycline’s potential clinical role in treating pulmonary infections.

## MATERIALS AND METHODS

A summary of the study procedure is provided in this section. Detailed description of routine procedures, statistical analysis and PK modeling techniques are available in the supplemental material.

To determine the PK/PD cutoff values for eravacycline, the PK/PD targets were first identified from preclinical studies. Then, human PK data (healthy volunteers) in plasma and ELF were obtained from a clinical trial and modeled using non-linear mixed-effect approach to evaluate drug exposure at the infection site (lungs). Using Monte Carlo simulations, the PTA across five dosing regimens was assessed to identify the MIC at which PTA was ≥90%, which was defined as the PK/PD cutoff.

### Bacterial strains

The MIC distribution of *E. coli*, *K. pneumoniae*, *S.aureus*, and *A. baumannii* was investigated. A total of 500 isolates were used for each bacterium in the phenotypic antimicrobial susceptibility testing (AST). The isolates were collected from the specimens (such as blood, sputum and bronchial secretions) of patients from five hospitals including the Peking Union Medical College Hospital, Peking University First Hospital (both in Beijing, China), Huashan Hospital, Fudan University (Shanghai, China), the First Affiliated Hospital of Guangzhou Medical University (Guangzhou, China), and Sir Run Run Shaw Hospital, Zhejiang University (Zhejiang, China). Please refer to Jing et al. for further details (39).

### *In vitro* antibacterial efficacy

MICs were determined using the twofold broth microdilution method described in the CLSI guidelines (9). The final test bacterial concentration was approximately 5 × 10^5^ CFU/mL, and the reaction volume was 0.1 mL. *E. coli*, *K. pneumoniae*, *S. aureus*, and *A. baumannii* were treated with eravacycline at concentrations ranging from 0.03 to 2, 0.03 to 16, 0.004 to 1, and 0.004 to 4 mg/L, respectively. The results of antibacterial experiments *in vitro* are listed in Table 2. For each bacterium, 90% inhibition of the tested bacterial isolates (MIC_90_) was selected as the threshold.

### Determination of PK/PD target values

Typically, bactericidal targets (1 or 2 log_10_ reduction) are preferred for antimicrobial agents targeting pulmonary infections, but a lower bacteriostatic threshold may be necessary for many tetracyclines (including eravacycline) which generally exhibits bacteriostatic activity (40). In both immunocompetent (10) or neutropenic (11) murine thigh infection models, eravacycline demonstrated some bactericidal activity against certain strains of *E. coli* but the bacterial-killing effect (i.e., 1-log_10_ reduction target) was not reached for *K. pneumoniae* or *Enterobacter cloacae* strains (10). Findings from these observations had been taken into consideration when the PK/PD target values were determined in this study.

Thabit et al. demonstrated that, in an immunocompetent murine thigh infection model, the median (range) *f*AUC/MIC required to achieve the stasis stage in four *E. coli* isolates was 2.41 (0.96–6.7), while to achieve a 1-log reduction, the required level was 8.5 (2.2–12.98; the endpoint was not reached in one isolate) (10). In addition, the published PK/PD cutoff value of eravacycline against *E. coli* is 0.5 mg/L according to the U.S. FDA (25), and the EUCAST has recommended a dosing regimen of 1 mg/kg q12h (41). The corresponding *f*AUC is 1.5 mg·h/L (8); hence, it is reasonable to infer that the target of eravacycline against *E. coli* would be *f*AUC/MIC = 3.

For *K. pneumoniae*, a preclinical *in vivo* study suggested that the *f*AUC/MIC of eravacycline that could result in bacteriostatic effects was 8.37 (10). In a mice study tested with *Enterobacteriaceae* strains, the bacteriostatic effect was demonstrated at *f*AUC/MIC = 6 and 6.8 (10). These results suggested that there was a large variation in the *in vivo* efficacy for *K. pneumoniae*. In the current study, the *f*AUC/MIC target for *K. pneumoniae* was set at a higher value of 10 as a conservative approach.

Concurrently, there was no published preclinical *in vivo* efficacy study against *A. baumannii* to provide a reference target value. But a Phase II/III clinical trial on patients with cIAI with primarily *A. baumannii* infection indicated that the median *f*AUC/MIC of eravacycline was 3, with a range from 1 to 12 (42). Due to the significant variation in the target value, it was decided that the *f*AUC/MIC target for *A. baumannii* would be set at 10 as a conservative but reasonable approach.

For *S. aureus*, the preclinical *in vivo* efficacy study indicated that the *f*AUC/MIC of eravacycline against methicillin-resistant *Staphylococcus aureus* that resulted in bacteriostatic effect was 9.6 (43). As such, the target value against *S. aureus* was determined at 10 in this study. All predetermined target values of eravacycline are summarized in the supplemental material (see Table S5).

### Development of population pharmacokinetics models

The blood and BAL concentrations data of eravacycline (1 mg/kg q12h) were extracted from a Phase I clinical trial in healthy volunteers (TP-434-006, NCT01989949). As this is a secondary analysis (8), no ethics clearance is required.

A total of 20 subjects (13 males) were included in the trial and their demographic data are presented in Table 5. The population PK model of eravacycline in both plasma and ELF was developed according to standard procedure. Briefly, a three-compartment structural PK model was developed using the abovementioned trial data. A fourth compartment representing pulmonary ELF concentration was established, and both plasma and BAL eravacycline concentrations were used to estimate unbound drug concentration in ELF. Allometric scaling was introduced into the final model. Subsequently, Monte Carlo simulations were performed for five dosing regimens (see Table 6) against all four tested bacteria for 7 days to determine PK/PD breakpoints and calculate the PTA and CFR. Detailed description is outlined in the supplemental material.

**TABLE 5:** Demographic characteristics of trial subjects^*a*^

Demographic characteristic (***n*** = 20)	Value
Sex*: male/female	13/7
Ethnicity*: Caucasian/others	15/5
Age (years)	27.5 [19.0, 47.0]
Height (cm)	170 [145, 191]
Body weight (kg)	81.4 [49.4, 105]
Red blood cell count (10^12^ /L)	26.8 [17.9, 35.3]
White blood cell count (10^9^ /L)	4.81 [3.80, 5.68]
Serum creatinine (mg/dL)	6.80 [4.30, 11.9]
Creatinine clearance (mL/min)	125.7 [73.22, 173.71]
Serum creatinine (mg/dL)	0.900 [0.600, 1.10]
Total protein (g/dL)	4.90 [4.70, 5.50]
Alanine aminotransferase, ALT (U/L)	7.40 [6.80, 8.00]
Aspartate aminotransferase, AST (U/L)	24.0 [11.0, 41.0]

^*a*^ The unit of measurement for variables annotated with an asteriak (*) is count number; that for the remaining variables is median [min, max].

**TABLE 6:** Five dosing regimens of eravacycline used in Monte Carlo simulation

No.	Dosing regimen	Remarks
1	1 h infusion, 1.0 mg/kg q12h for 7 days	Approved dosage
2	2 h infusion, 1.5 mg/kg q12h for 7 days	Proposed high dosage
3	2 h infusion, 1.5 mg/kg q24h for 7 days	Proposed low dosage
4	2 h infusion, 1.5 mg/kg q12h on first day, 1.5 mg/kg q24h second day onwards	Proposed low dosage
5	2 h infusion, 1.5 mg/kg q12h on first day, 1.0 mg/kg q12h second day onwards	Proposed high dosage

## SUPPLEMENTAL MATERIAL

Supplemental material aac.01065-24-s0001.docx Supplemental methods, Tables S1 to S5, and Figures S1 to S7.

ASM does not own the copyrights to Supplemental Material that may be linked to, or accessed through, an article. The authors have granted ASM a non-exclusive, world-wide license to publish the Supplemental Material files. Please contact the corresponding author directly for reuse.
