# Supplementary material for: Population pharmacokinetics and pulmonary modeling of eravacycline and the determination of microbiological breakpoint and cutoff of PK/PD
Source: Antimicrob Agents Chemother. 2025 Jan 29;69(3):e01065-24. doi: 10.1128/aac.01065-24 (PMC11881576; doi:10.1128/aac.01065-24)
Supplement: Supplemental material — Supplemental methods, Tables S1 to S5, and Figures S1 to S7. [file aac.01065-24-s0001.docx]

**Supplementary Material**

1. **Materials and Methods**
   1. **Bioanalytical Assay**

An LC/MS/MS bioanalytical method for the quantitation of eravacycline in human BAL fluid and lysed macrophage fractions was validated using saline as the surrogate matrix. In this method, eravacycline was fortified into saline (or BAL fluid) at 10 μg/mL and sequentially diluted with saline (or BAL fluid) to prepare calibration curve standards and quality control (QC) samples. Sample volume was 100 μL and the injection volume was 5 μL. An AB Sciex 5500 LC/MS/MS system was used for the analysis. The method is selective and reproducible, and showed matrix equivalency of BAL fluid and saline. The summary validation data are presented below:

| Analytes | eravacycline (TP-434) |
| --- | --- |
| Internal standards | TP-434-d_8_ |
| Calibration range | 5-500 ng/ml |
| Dilution verification | 10-fold |
| Regression type | Liner, weight (1/x^2^) |
| Sample volume | 100 μl of human BAL fluid or saline |
| Intra-day precision and accuracy | %CV: 2.7%-7.6%; %Bias: 1.2%-9.0% (for LLOQ)  %CV: 2.8%-6.4%; %Bias: -6.0%-5.5% (for other QC levels) |
| Inter-day precision and accuracy | %CV: 5.5%; %Bias: 5.4% (for LLOQ)  %CV: 4.1%-5.8%; %Bias: -2.0%-0.3% (for other QC levels) |
| Selectivity | No significant interferences observed |
| Carry over | No significant interferences observed |
| Matrix equivalency | No obvious difference observed between saline and BAL fluid |
| Recovery from saline | 97.6%-105.4% |
| Bench-top stability in saline | At least 6 hours at 4℃ |
| Freeze/Thaw stability | At least 3 cycles |
| Extract storage stability | At least 67 hours at 4℃ |
| Extract autosampler stability | At least 94 hours at 5℃ |
| Frozen sample long-term storage stability | At least 25 days at -20℃ and -70℃ |

1. **Development of population pharmacokinetics models**
   1. **Human PK data collection**

The blood and bronchoalveolar lavage (BAL) concentrations data of eravacycline were extracted from a phase Ⅰ clinical trial in healthy volunteers (TP-434-006, NCT 01989949). A total of 20 subjects (13 males) were included in the trial and their demographic data are presented in Table 1. All study participants received seven intravenous infusions (given over 1h) of eravacycline 1mg/kg q12h. Blood samples were collected prior to drug administration (0h) and at 1, 2, 4, 6, and 12h post-administration of the last dose. BAL samples were collected at 1, 2, 4, 6, and 12h after the last dose via bronchoscopy.^1^

Information on eravacycline protein binding was based on published data.^1, 2^ Briefly, the relationship between total drug concentration and protein binding was nonlinear and a power function was used to calculate the bound concentration of eravacycline. In turn, the conversion of total plasma concentration to free plasma concentration was performed via equation (1) below:

$C_{u}=0.141\cdot C_{tot}^{0.873}$ (1)

where $C_{u}$ was the free plasma drug concentration, and $C_{tot}$ was the total drug concentration.

- 1. **ELF concentration determined from BAL**

As concentrations in ELF could not be measured directly, these were estimated with BAL. To correlate eravacycline concentrations between BAL and ELF, a series of conversions were performed following equations (2) to (4):

$V_{ELF}=V_{BAL}\times\frac{urea_{BAL}}{urea_{plasma}}$ (2)

$C_{ELF}=C_{BAL}\times\frac{V_{BAL}}{V_{ELF}}$ (3)

$C_{ELF}=C_{BAL}\times\frac{urea_{plasma}}{urea_{BAL}}$ (4)

where $V_{ELF}$ and $V_{BAL}$ were the volumes of eravacycline in ELF and BAL, $C_{ELF}$ and $C_{BAL}$ were eravacycline concentrations in ELF or BAL, $urea_{plasma}$ and $urea_{BAL}$ were urea concentrations in plasma or BAL. The concentration of protein, such as albumin, in ELF had been reported to be significantly lower than plasma level.^3^ As such, protein binding of eravacycline in ELF was expected to be negligible. Furthermore, only free drug molecules could be transported freely between the plasma and ELF, hence the calculation of free eravacycline concentration in ELF did not consider protein binding.

- 1. **Structural model**

Conventional compartmental PK models were tested sequentially. Model selection was based on changes in the Akaike information criterion (AIC), the precision of parameter estimates (relative standard errors, RSE) and standard goodness-of-fit (GoF) plots.^4^ Eventually, the following 3-compartment structural model was fitted to the drug concentration of eravacycline in plasma and ELF. The model equations were:

$\frac{dA_{1}}{dt}=-k_{e}A_{1}-k_{12}A_{1}+k_{21}A_{2}-k_{13}A_{1}+k_{31}A_{3}-k_{cl}A_{1,free}+k_{lc}A_{ELF}$(5)

$\frac{dA_{2}}{dt}=k_{12}A_{1}-k_{21}A_{2}$ (6)

$\frac{dA_{3}}{dt}=k_{13}A_{1}-k_{31}A_{3}$ (7)

$\frac{dA_{ELF}}{dt}=k_{cl}A_{1,free}-k_{lc}A_{ELF}$ (8)

$A_{1,free}=V_{C}C_{u}=V_{C}\times0.141\times C_{tot}^{0.873}$ $=V_{C}\times0.141\times{(\frac{A_{1}}{V_{C}})}^{0.873}$ (9)

Equations 5-9 described the rate of change of the amount of eravacycline (represented by $A_{1}, A_{1,free}, A_{2}, A_{3}$ and $A_{ELF}$ variables) in the central (both total and free), first and second peripheral compartment, and the ELF compartment respectively; $k_{e}$ was the eliminate rate constant, $k_{cl}$ and $k_{lc}$ were the first-order intercompartmental clearance rate constant between the central and ELF compartment, while the remaining $k$-variables with the appropriate subscripts represent the first-order intercompartmental rate constant.

Since the BAL samples used to estimate eravacycline concentration in the ELF compartment were sparse and all the BAL samples were collected after reaching steady state, the resultant estimation of ELF-related kinetic parameters could be imprecise. To overcome this shortcoming, the model would be simplified based on the assumption that the concentration between the central and ELF compartment had reached a quasi-equilibrium status. Hence, the following equations could be derived:

$\frac{dA_{ELF}}{dt}=k_{cl}A_{1, free}-k_{lc}A_{ELF}=0$ (10)

$A_{ELF}=\frac{{k_{cl}A}_{1,free}}{k_{lc}}$ (11)

Converting eravacycline mass to concentration:

$C_{ELF}=\frac{A_{ELF}}{V_{ELF}}=\frac{A_{1,free}k_{cl}}{k_{lc}V_{ELF}}=\frac{C_{u} V_{C}k_{cl}}{V_{ELF}k_{lc}}$ $=C_{u}\times Ratio$ (12)

where $C_{ELF}$ and $C_{u}$ were the unbound eravacycline concentration in the ELF and central compartment, $V_{ELF}$ and $V_{c}$ were the volume of distribution of the ELF and central compartment. The fraction $\frac{V_{c} k_{cl}}{V_{ELF} k_{lc}}$ was a constant and could be represented by $Ratio$.

- 1. **Statistical model**

*Interindividual variability*

Interindividual variability (IIV) terms were added to the structural model in an exponential manner, as shown in equation (10) below:

$P_{i}=\theta\cdot e^{\eta_{i}}$ (13)

where $P_{i}$ was the individual parameter estimate, $\theta$ is the population estimate of the parameter, and $\eta_{i}$ is the deviation from the population estimate for the i^th^ individual which was assumed to be normally distributed with a mean of zero and variance of $\omega^{2}$. Covariance between clearance and volume parameters was tested.

*Residual variability*

Residual variability (RSV) was included to ensure the individual weighted residuals (IWRES) were approximately homoscedastically distributed across all predictors.

*Covariate model*

A graphical exploratory analysis of all collected covariates was conducted to examine their correlation. Univariate analyses of variance (ANOVA) were performed for categorical covariates while linear regression was used for continuous covariates.

The covariate *body weight* was included *a priori* as part of the structural model via allometric scaling. For other covariates, stepwise covariate modelling (SCM) technique was used to select relevant covariates through the forward inclusion and backward elimination steps. The final decision for covariate inclusion was based on both statistical evidence as well as clinical knowledge. Continuous covariates were added into the model via a linear or power function as shown in Equations 14-15 below:

$P_{j}=\theta_{0}+\theta_{1}\cdot\left( X_{1}-Median\left( X_{1} \right) \right)$ (14)

$P_{j}=\theta_{0}\cdot\left( \frac{X_{1}}{Median\left( X_{1} \right)} \right)^{\theta_{1}}$ (15)

where $P_{j}$ denoted the $j^{th}$ parameter, $\theta_{0}$ was the intercept, $\theta_{1}$ was the slope associated with the covariates, $X_{1}$ was the covariates, ${Median(X}_{1})$ was the median of covariate $X_{1}$.

For dichotomous covariates, these were added linearly as shown by Equation 16.

$P_{j}=\theta_{0}\cdot\left( 1+\theta_{1}\cdot X_{1} \right)$ (16)

*Model evaluation*

The final model was evaluated graphically on GoF plots, including observed values versus individual prediction or population prediction, conditional weighted residuals (CWRES) versus TIME, absolute individual weighted residuals (|IWRES|) versus individual predictions, and the normality test of CWRES.

Bootstrap was performed to internally validate the final model. Median values and 95% confidence intervals (CI) of 1000 resampling were calculated and compared with the final model parameter estimates to assess the robustness of the final model.

Visual predictive checks (VPCs) were used to evaluate the predictive power of the final model. One thousand simulations were implemented and the 5^th^, 50^th^, and 95^th^ percentiles of the observed versus simulated data were compared.

*Dose optimization via Monte Carlo simulations*

Eravacycline concentrations following five different dosing regimens (see Table 2) were simulated against all four tested bacteria for seven days, based on the final population PK model estimates where the virtual subjects’ body weight followed a uniform distribution between 50-100kg. The ratios of free eravacycline AUC at steady state over MIC values (*f*AUC_SS_/MIC) were calculated. Based on predefined PK/PD targets, the PTA for each investigated MIC was determined based on Equation 17 below:

$\mathrm{PTA}\left( \% \right)=\frac{\sum_{i=1}^{n} f\left( \frac{\mathrm{AUC}}{MIC_{i}} \right)}{n}\cdot100\%$ (17)

where $n$ was the total number of $i^{th}$ subjects; $\frac{AUC}{MIC_{i}}$ was the concentration-dependent PK/PD index for the $i^{th}$ subjects. The function $f(x)$ was a logical function where it would return 1 when $\frac{AUC}{MIC_{i}}$ was larger or equal to the target, and return 0 when $\frac{AUC}{MIC_{i}}$ was less than the target as Equation 18 below:

$f\left( x \right)=\left\{ \begin{aligned} 1, &x\geq target \\ 0, &x<target \end{aligned} \right.$ (18)

The PK/PD breakpoint was defined as the MIC value that corresponded to PTA=90%. The resultant cumulative fraction of response (CFR) was obtained through Equation 19:

$CFR\left( \% \right)=\sum_{j=1}^{m} {PTA}_{j}\cdot{Frac}_{MIC,j}$ (19)

where $m$ was the total number of MIC tested for each bacterium, $j$ was the $j^{th}$ number of MIC tested, $PTA_{J}$ was the probability of attaining the PK/PD target given the $j^{th}$ MIC, $Frac_{MIC,j}$ was the fraction of the isolate that demonstrated the MIC value that corresponded with the MIC value of the $j^{th}$ isolate population.

- 1. **Handling of missing, below-quantitative-limit (BQL), and outlier data**

When the proportion of below-the-quantification limit (BQL) data was larger than 10%, the likelihood-based approach (such as the M3 method) along with Laplacian estimation was used.^5, 6^ Observations corresponded to absolute conditional weighted residuals of more than 5 (|CWRES|>5) were regarded as outliers and omitted from the model-building process^7^. These omitted points were re-introduced during sensitivity analyses to assess their impact on parameter estimates.

- 1. **Software details**

The population PK model was developed with NONMEM (version 7.5, ICON Development Solutions, MD, USA). First order conditional estimation with interaction (FOCEI) method was used for parameter estimation.^8^ R (version 3.5.3) was used for data preparation, graphical analysis, model diagnostics, and statistical summaries. Xpose^®9^ and Pearl Speaks NONMEM (PsN®)^4, 10^ were used for model diagnostics and to facilitate other model development tasks.

Table S1: Key model development steps

| Model number | Reference model | AIC | ΔAIC | Remark |
| --- | --- | --- | --- | --- |
| 1 | / | 1408 | / | 1-cmt, kinetic lung distribution |
| 2 | 1 | 1177 | -231 | 2-cmt, kinetic lung distribution |
| 3 | 1 | 1174 | -234 | 3-cmt, kinetic lung distribution |
| 4 | 1 | 1190 | -218 | 2-cmt, quasi-steady lung distribution |
| 5 | 3 | 1170 | -4 | 3-cmt, quasi-steady lung distribution |
| 6 (final) | 5 | 1156 | -14 | 3-cmt, quasi-steady lung distribution, weight added allometrically |

Abbreviation: AIC, Akaike information criterion; ΔAIC, changes in AIC; cmt, compartment; ELF, epithelial lining fluid

Table S2: covariate selection steps

**FORWARD SELECTION**

| **MODEL** | **OFV** | **DROP in OFV** | **P-value** | **Decision** |
| --- | --- | --- | --- | --- |
| **Forward inclusion (round 1)** | | | | |
| Base | 1130.33196 | - | - |  |
| **QSEX-2** | **1121.15373** | **9.17823** | **0.002449** | **Include** |
| Chosen model with QSEX-2  Model OFV = 1121.15373 | | | | |

| **Forward inclusion (Round 2)** | | | | |
| --- | --- | --- | --- | --- |
| Base | 1121.15373 |  |  |  |
| **CLALT-2** | **1110.37968** | **10.77405** | **0.001029** | **Include** |
| **Q2RBC-2** | **1112.51022** | **8.64350** | **0.003282** | **Include** |
| **VPRBC-2** | **1113.39813** | **7.75560** | **0.005355** | **Include** |
| Chosen model with CLALT-2 and QSEX-2  Model OFV = 1110.37968 | | | | |

| **Forward inclusion (Round 3)** | | | | |
| --- | --- | --- | --- | --- |
| Base | 1110.37968 |  |  |  |
| No covariate was included. Forward search done | | | | |
|  |  |  |  |  |

**BACKWARD ELIMINATION**

| **Backward Elimination (Round 1)** | | | | |
| --- | --- | --- | --- | --- |
| Base | 1110.37968 |  |  |  |
| CLALT-1 | 1121.15373 | -10.77405 | 0.001029 | Exclude |
| QSEX-1 | 1125.65740 | -15.27772 | 0.000093 |  |
|  |  |  |  |  |
| Chosen model with QSEX-1 | | | | |
| Model OFV = 1121.15373 | | | | |

| **Backward Elimination (Round 2)** | | | | |
| --- | --- | --- | --- | --- |
| Base | 1121.15373 |  |  |  |
| QSEX-1 | 1130.30306 | -9.14933 | 0.002488 | Exclude |
|  |  |  |  |  |

Note: The stepwise covariate modeling (SCM) procedure had systematically evaluated the effects of eight covariates on the model parameters. These covariates included *age, albumin, alanine aminotransferase, creatinine clearance, total protein, red blood cells, sex,* and *white blood cells*. Due to space constrains in the table, for each forward selection round, only the covariates that satisfied the inclusion criteria were listed.

Table S3: Reported values of eravacycline clearance

| **Source** | **Study Number** | **Reported CL (unit of measurement)** | **Reported mean weight (kg)** | **Weight adjusted CL* (L/h/kg)** |
| --- | --- | --- | --- | --- |
| Xerava NDA | TP-434-P1-MAD-1  (1mg/kg BID, day 1 values) | 0.18 L/h/kg | NR | 0.18 |
|  | TP-434-026  (1mg/kg single dose) | 3.90 ml/min/kg | NR | 0.23 |
|  | TP-434-020  (1mg/kg single dose) | 3.20 ml/min/kg | NR | 0.19 |
| Conor et al 2014 | - | 17.82 L/h | 80 | 0.22 |
| Newman et al 2018 | SAD 1mg/kg | 0.19 L/h/kg | 80.16 | 0.19 |
|  | MAD 1mg/kg q12h | 0.16 L/h/kg | 79.3 | 0.16 |
| Newman et al 2019 | 1mg/kg single dose | 228 ml/min | 87.1 | 0.16 |
|  | 1mg/kg DDI (itraconazole, day 1 value) | 3.79 ml/min/kg | 69.7 | 0.23 |
|  | 1mg/kg DDI (rifampicin, day 1 value) | 3.20 ml/min/kg | 77.5 | 0.19 |
| Current study | 1mg/kg q12h | 16.3 L/h | 81.4 | 0.20 |

NR: not reported.

*When weight was not reported, the value of 80kg was used

Table S4: Predetermined target values of eravacycline against different tested bacterium.

| Species | Target values |
| --- | --- |
| *E. coli* | 3 |
| *K. pneumoniae* | 3, 4, 6, 8, 10 |
| *A. baumannii* | 3, 4, 6, 8, 10 |
| *S. aureus* | 2, 4, 6, 8, 10 |

Table S5(A): Corresponding PTA values at each level of MIC for *E.coli* under different dosing regimens

*f*AUC/MIC=3

| **PTA（%）** | | | | | |
| --- | --- | --- | --- | --- | --- |
| **MIC (mg/L)** | **Regimen 1** | **Regimen 2** | **Regimen 3** | **Regimen 4** | **Regimen 5** |
| 0.03 | 100 | 100 | 100 | 100 | 100 |
| 0.06 | 100 | 100 | 100 | 100 | 100 |
| 0.12 | 100 | 100 | 100 | 100 | 100 |
| 0.25 | 100 | 100 | 100 | 100 | 100 |
| 0.5 | 100 | 100 | 100 | 100 | 100 |
| 1 | 100 | 100 | 100 | 100 | 100 |
| 2 | 100 | 100 | 99.9 | 100 | 100 |
| 4 | 63.3 | 100 | 3.1 | 4.2 | 60.8 |
| 8 | 0 | 1 | 0 | 0 | 0 |
| 16 | 0 | 0 | 0 | 0 | 0 |
| **CFR（%）** | **100** | **100** | **100** | **100** | **100** |

Table S5(B): Corresponding PTA values at each level of MIC for *K.pneumoniae* under different dosing regimens

*f*AUC/MIC=10

| **PTA（%）** | | | | | |
| --- | --- | --- | --- | --- | --- |
| **MIC (mg/L)** | **Regimen 1** | **Regimen 2** | **Regimen 3** | **Regimen 4** | **Regimen 5** |
| 0.03 | 100 | 100 | 100 | 100 | 100 |
| 0.06 | 100 | 100 | 100 | 100 | 100 |
| 0.12 | 100 | 100 | 100 | 100 | 100 |
| 0.25 | 100 | 100 | 100 | 100 | 100 |
| 0.5 | 100 | 100 | 100 | 100 | 100 |
| 1 | 95.1 | 100 | 38.2 | 38.2 | 95.9 |
| 2 | 0 | 18.4 | 0 | 0 | 0 |
| 4 | 0 | 0 | 0 | 0 | 0 |
| 8 | 0 | 0 | 0 | 0 | 0 |
| 16 | 0 | 0 | 0 | 0 | 0 |
| **CFR (%)** | **87.8** | **89.6** | **82.8** | **82.8** | **87.8** |

*f*AUC/MIC=6

| **PTA（%）** | | | | | |
| --- | --- | --- | --- | --- | --- |
| **MIC (mg/L)** | **Regimen 1** | **Regimen 2** | **Regimen 3** | **Regimen 4** | **Regimen 5** |
| 0.03 | 100 | 100 | 100 | 100 | 100 |
| 0.06 | 100 | 100 | 100 | 100 | 100 |
| 0.12 | 100 | 100 | 100 | 100 | 100 |
| 0.25 | 100 | 100 | 100 | 100 | 100 |
| 0.5 | 100 | 100 | 100 | 100 | 100 |
| 1 | 100 | 100 | 99.9 | 100 | 100 |
| 2 | 63.3 | 100 | 3.1 | 4.2 | 60.8 |
| 4 | 0 | 1 | 0 | 0 | 0 |
| 8 | 0 | 0 | 0 | 0 | 0 |
| 16 | 0 | 0 | 0 | 0 | 0 |
| **CFR (%)** | **93.0** | **95.8** | **88.4** | **88.5** | **92.8** |

Table S5(C): Corresponding PTA values at each level of MIC for *A.baumannii* under different dosing regimens

*f*AUC/MIC=10

| **PTA（%）** | | | | | |
| --- | --- | --- | --- | --- | --- |
| **MIC (mg/L)** | **Regimen 1** | **Regimen 2** | **Regimen 3** | **Regimen 4** | **Regimen 5** |
| 0.004 | 100 | 100 | 100 | 100 | 100 |
| 0.008 | 100 | 100 | 100 | 100 | 100 |
| 0.016 | 100 | 100 | 100 | 100 | 100 |
| 0.03 | 100 | 100 | 100 | 100 | 100 |
| 0.06 | 100 | 100 | 100 | 100 | 100 |
| 0.12 | 100 | 100 | 100 | 100 | 100 |
| 0.25 | 100 | 100 | 100 | 100 | 100 |
| 0.5 | 100 | 100 | 100 | 100 | 100 |
| 1 | 95.1 | 100 | 38.2 | 38.2 | 95.9 |
| 2 | 0 | 18.4 | 0 | 0 | 0 |
| 4 | 0 | 0 | 0 | 0 | 0 |
| 8 | 0 | 0 | 0 | 0 | 0 |
| 16 | 0 | 0 | 0 | 0 | 0 |
| **CFR (%)** | **97.4** | **97.9** | **94.9** | **94.9** | **97.4** |

Table S5(D): Corresponding PTA values at each level of MIC for *S.aureus* under different dosing regimens

*f*AUC/MIC=10

| **PTA（%）** | | | | | |
| --- | --- | --- | --- | --- | --- |
| **MIC (mg/L)** | **Regimen 1** | **Regimen 2** | **Regimen 3** | **Regimen 4** | **Regimen 5** |
| 0.004 | 100 | 100 | 100 | 100 | 100 |
| 0.008 | 100 | 100 | 100 | 100 | 100 |
| 0.016 | 100 | 100 | 100 | 100 | 100 |
| 0.03 | 100 | 100 | 100 | 100 | 100 |
| 0.06 | 100 | 100 | 100 | 100 | 100 |
| 0.12 | 100 | 100 | 100 | 100 | 100 |
| 0.25 | 100 | 100 | 100 | 100 | 100 |
| 0.5 | 100 | 100 | 100 | 100 | 100 |
| 1 | 95.1 | 100 | 38.2 | 38.2 | 95.9 |
| 2 | 0 | 18.4 | 0 | 0 | 0 |
| 4 | 0 | 0 | 0 | 0 | 0 |
| 8 | 0 | 0 | 0 | 0 | 0 |
| 16 | 0 | 0 | 0 | 0 | 0 |
| **CFR (%)** | **99.9** | **100** | **98.5** | **98.5** | **99.9** |


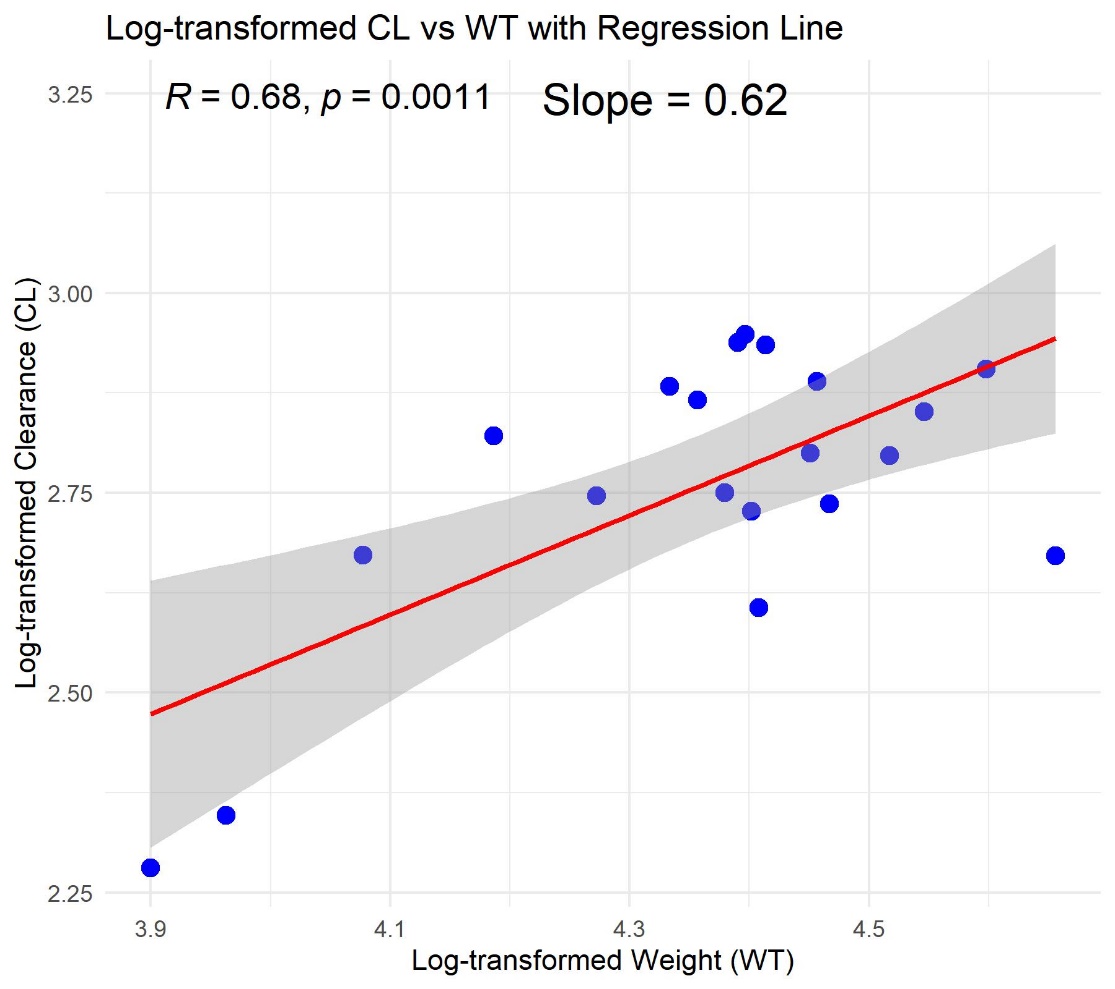


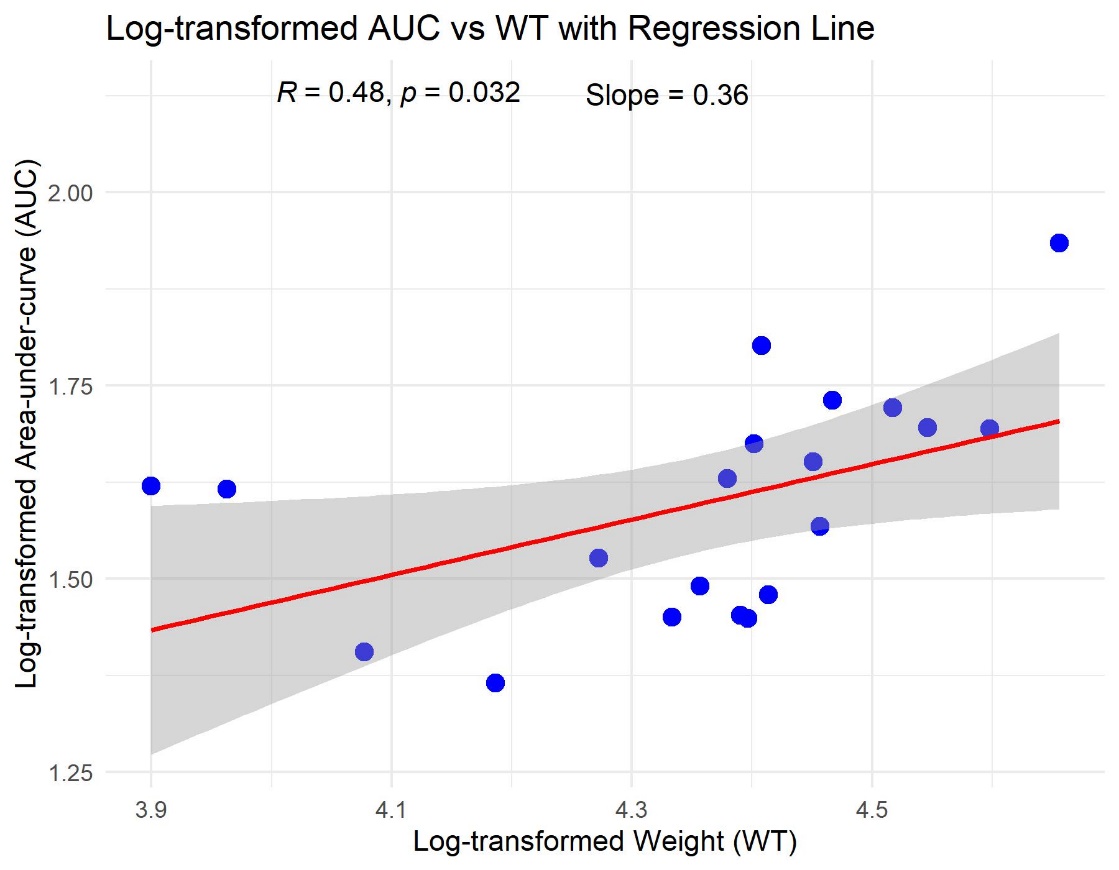


Figure S1: Correlation between weight and eravacycline clearance (top) and AUC (bottom)


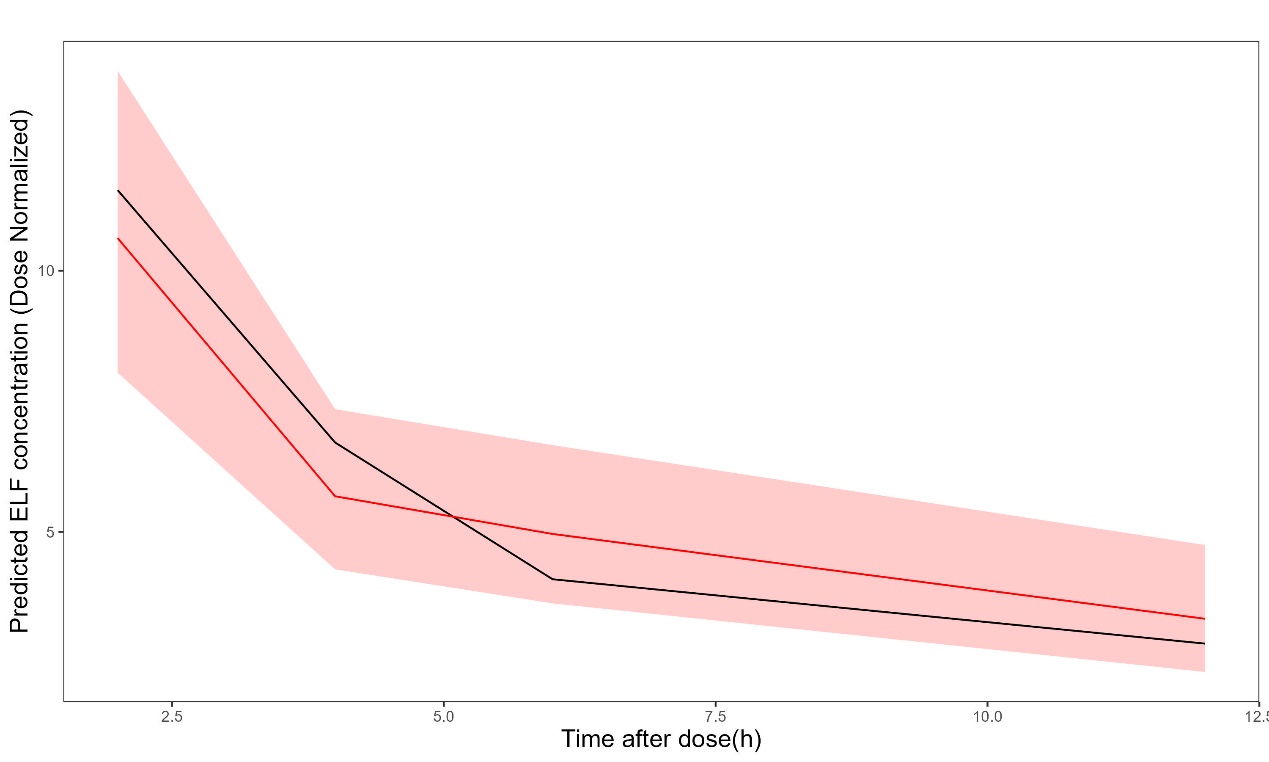


Figure S2: Visual predictive check of eravacycline ELF concentration. Legend: black solid line, median value of observed ELF concentration; red solid line, median value of predicted ELF concentration; red band, 90% prediction interval of ELF concentration


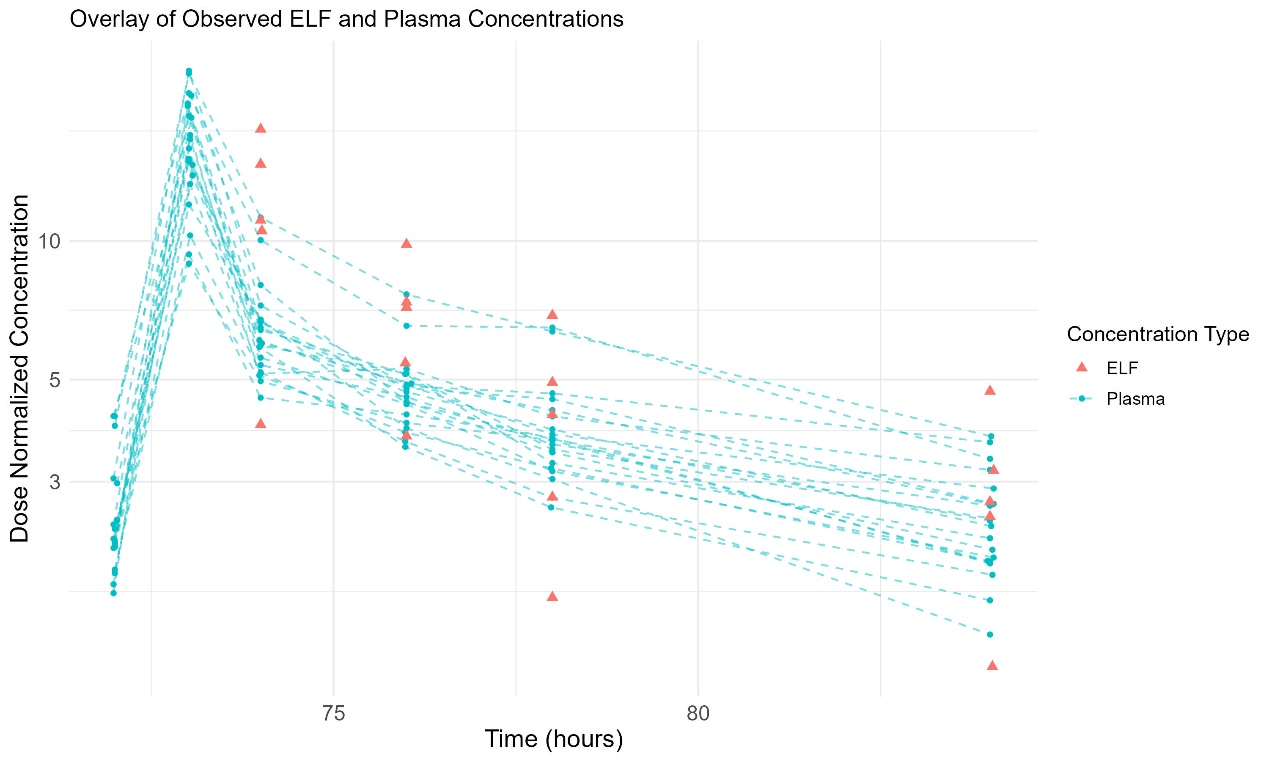


Figure S3: Dose normalized eravacycline concentration in plasma and ELF. Legend: Blue line or dot represents observed plasma concentration, red triangle represents ELF concentration.


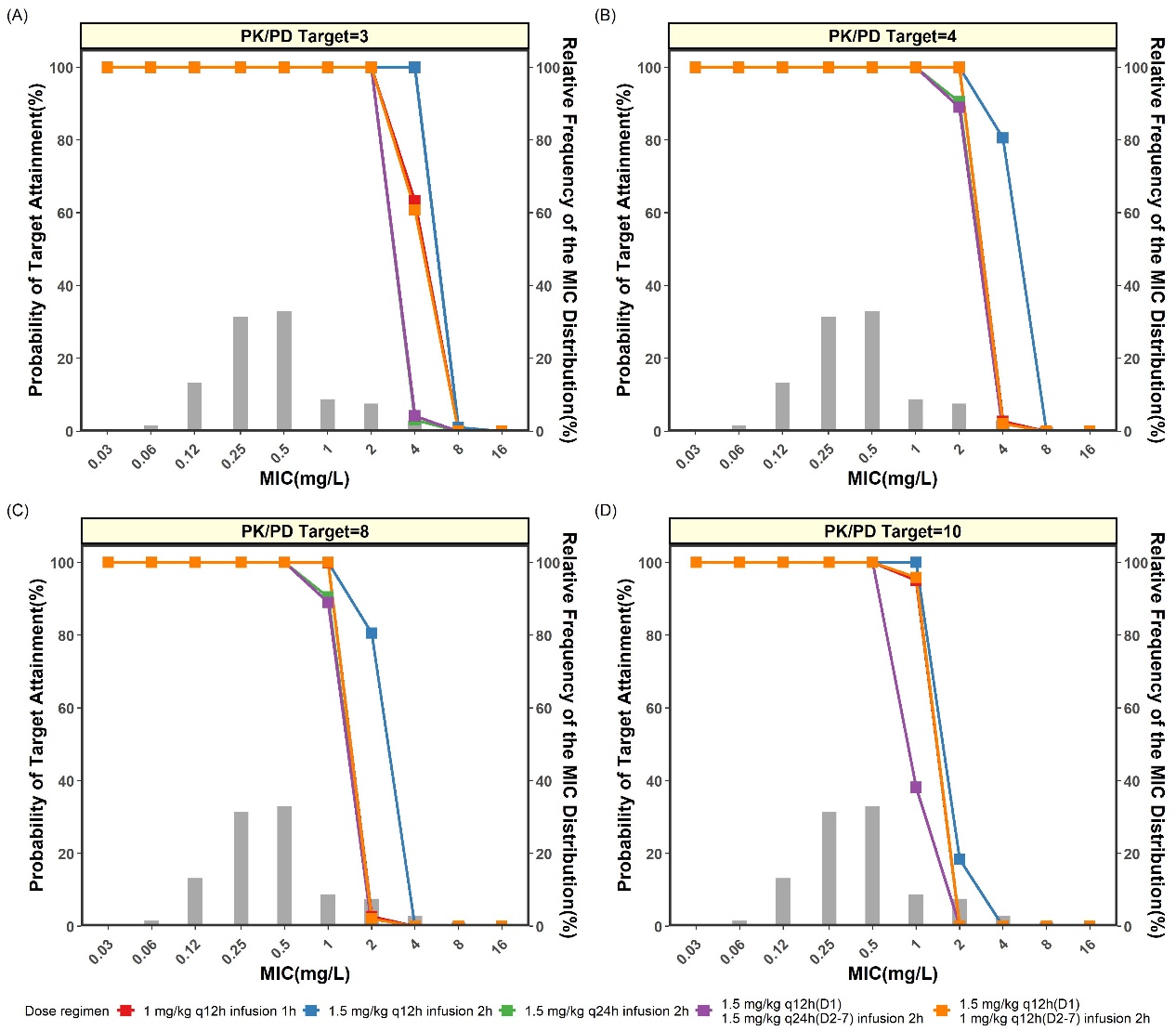


Figure S4: Probability of target attainment of eravacycline against *K. pneumonia* under different target values: (A) *f*AUC/MIC=3, (B) *f*AUC/MIC=4, (C) *f*AUC/MIC=8, and (D) *f*AUC/MIC=10


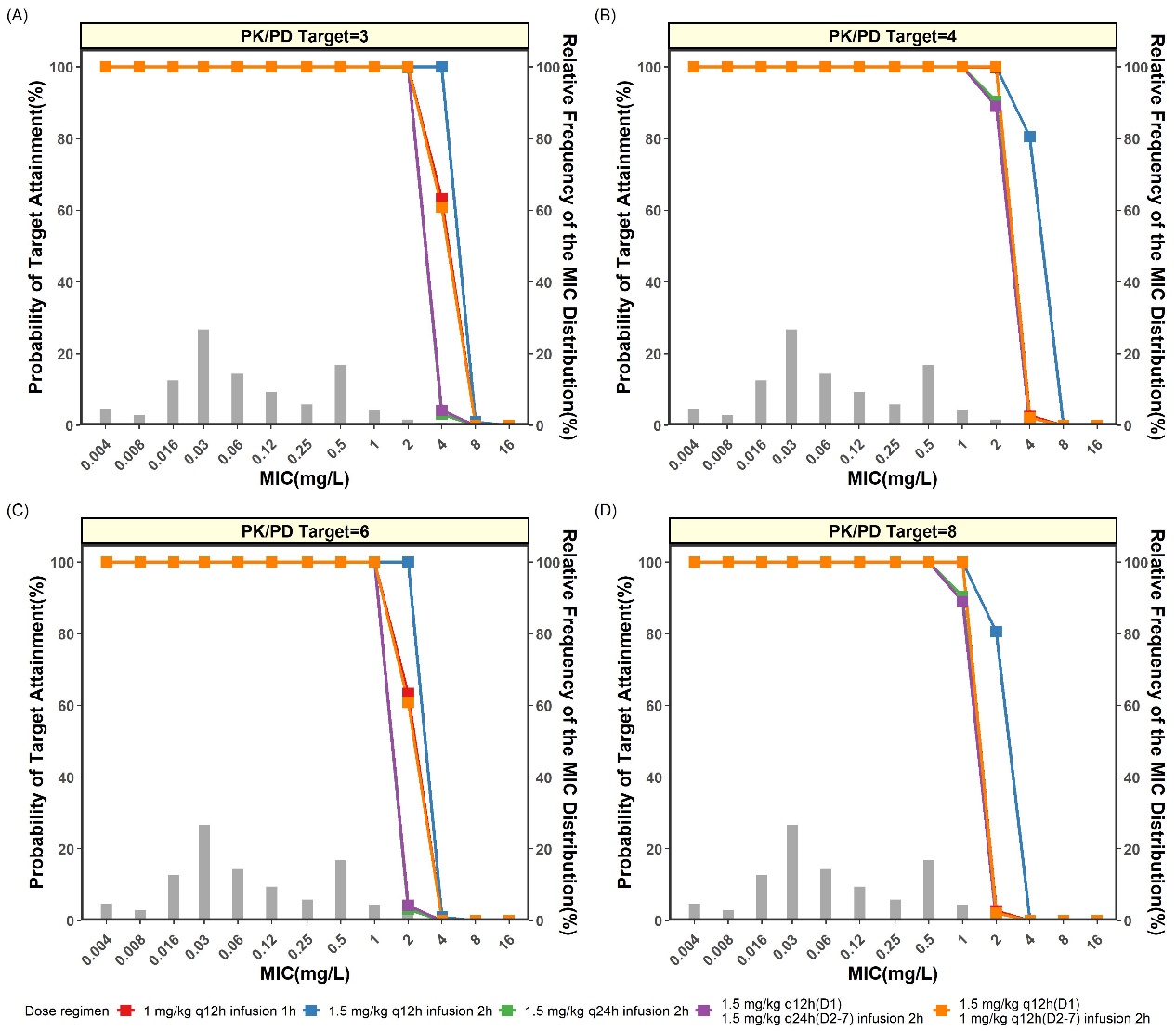


Figure S5: Probability of target attainment of eravacycline against *A. baumannii* under different target values: (A) *f*AUC/MIC=3, (B) *f*AUC/MIC=4, (C) *f*AUC/MIC=6, and (D) *f*AUC/MIC=8


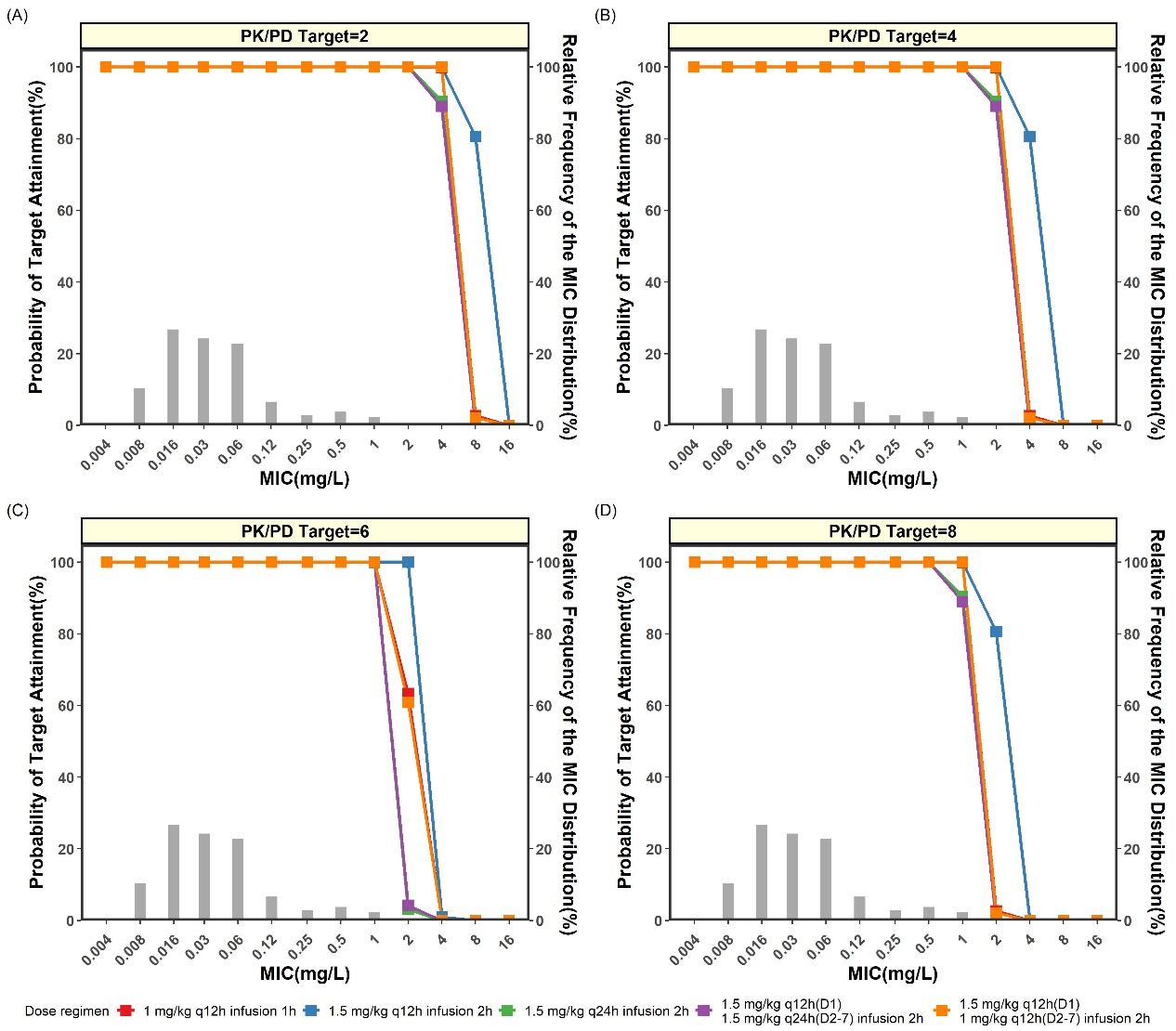


Figure S6: Probability of target attainment of eravacycline against *S. aureus* under different target values: (A) *f*AUC/MIC=2, (B) *f*AUC/MIC=4, (C) *f*AUC/MIC=6, and (D) *f*AUC/MIC=8


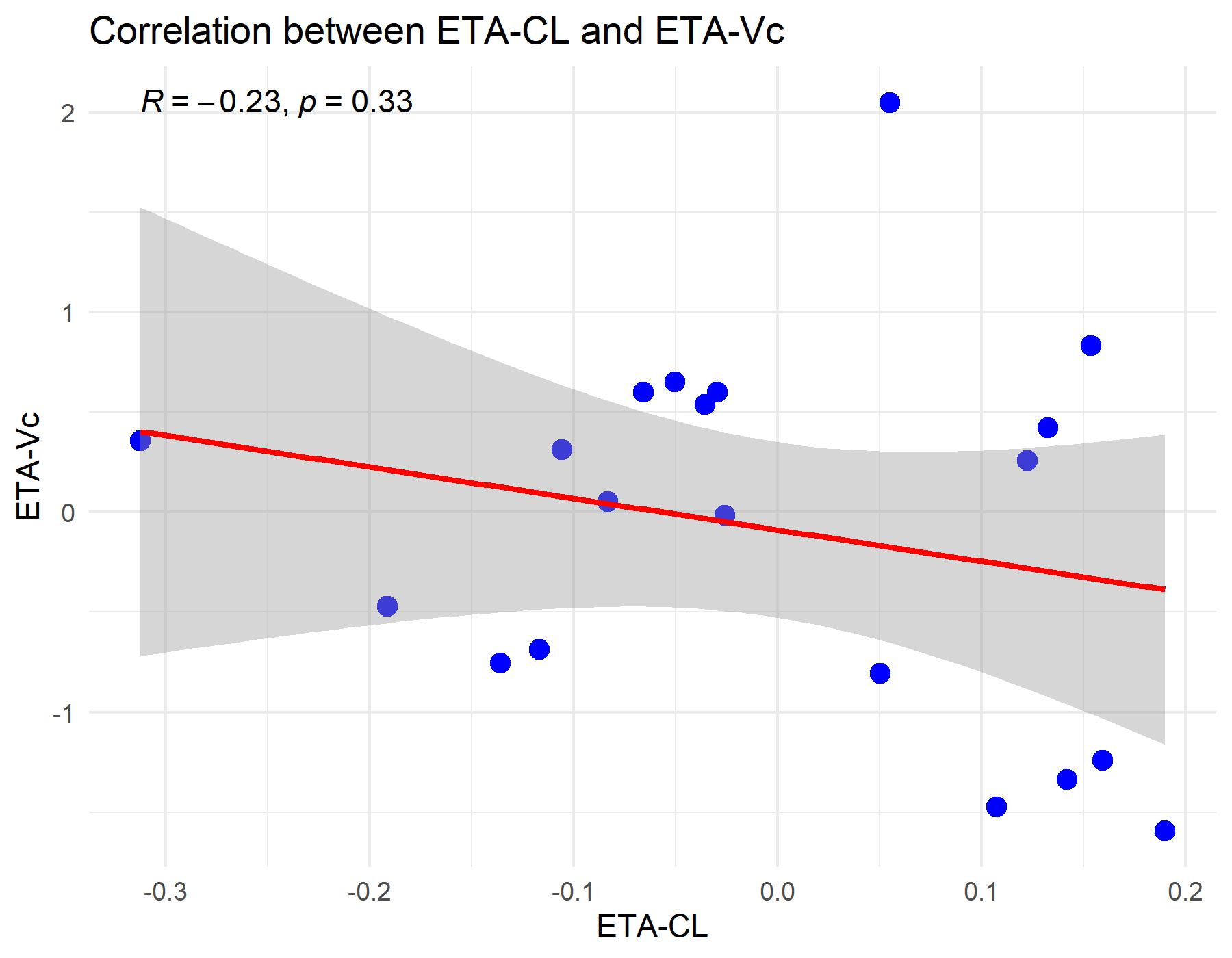


Figure S7: Correlation between ETA-CL and ETA-Vc

**Comprehensive evaluation project for clinical application of eravacycline**

**[English translation]**

There are 3369 cases (2192 males and 1177 females with an average age of 58.0±17.8 years) from 231 Chinese hospitals since September, 2023 to September, 2024 included in a comprehensive evaluation project for clinical application of eravacycline. This project was initiated and hosted by the expert committee on clinical use of antimicrobials and evaluation of antimicrobial resistance, national health commission of the people’s republic of China. Among these cases, the number of patients from the intensive care unit (ICU) was the highest, followed by hematology department, and the proportions are 46.8% and 23.6%, respectively. The other cases are from transplantation department, respiratory department, emergency department, and infectious disease department, etc.

The results of study indicated that eravacycline show good efficacy and safety in the treatment of pulmonary infection and bloodstream infection, besides abdominal infection. In this project, the most common type of infection is pulmonary infection, and the proportions is 62.5%. The invitro susceptibility tests suggested the sensitive rates of *Acinetobacter baumannii, Klebsiella pneumoniae, Escherichia coli,* and *Staphylococcus aureus* to eravacycline were 95.5%, 92.5%, 72.1% and 92.5% (according to ChinaCAST breakpoints), these to tigecycline were 77.2%, 75.6%, 88.0% and 81.8% in comparison (according to FDA breakpoints).

The pathogen distribution of this project is mainly composed of carbapenem resistant *Acinetobacter baumannii* and *Klebsiella pneumoniae*. The conditions of the patients included in the project are serious, and most of them suffered from basic diseases.

The total response rate of eravacycline was 90.1% at the end of treatment. The clinical effective rates of eravacycline in ICU, hematology department, transplantation department and infectious disease department were 88.7%, 88.8%, 94.4% and 95.5%, respectively. Results of pathogenic analysis suggested the therapeutical effective rates of eravacycline against *Acinetobacter baumannii* and *Klebsiella pneumoniae* were 91.4% and 91.2%, respectively. The microbiology response rate of eravacycline was 90.7% at the end of treatment. As the result of the therapeutic outcome on the 30th day, the total response rate of eravacycline was 85.9%, which were 86.0% and 86.7% against *Acinetobacter baumannii* and *Klebsiella pneumoniae*. The prognostic analysis showed the rate of improvement to hospital discharge after treatment with eravacycline was 83.0%.

The clinical study indicated that eravacycline has good safty. The total occurrence rate of adverse reaction was 2.7%.

In this project, 94.6% of doctors chose the standard dosage (1mg/kg, q12h) in the clinical treatment with eravacycline, and 4.3% of doctors chose the dosage of 50 mg/kg, q12h. The average course of treatment for standard dose therapy was 9 days.

The project results indicate that eravacycline show good antibacterial activity in the real world, and it is effective in the treatment of the patients with the infections caused by drug-resistant bacteria.

**[Original Mandarin Text]**

**依拉环素临床应用综合评价项目**

本项目共纳入2023年9月至2024年9月来自全国231家医院的3369例病例数据，其中男性2192例，女性1177例，平均年龄为58.0±17.8岁。来自重症监护病房（ICU）的患者最多，其次为血液科，分别占比46.8%和23.6%，其余病例来自移植科、呼吸科、急诊科及感染科等。

调研结果显示，依拉环素不仅能有效治疗腹腔感染，在肺部感染、血流感染等也显示出良好的疗效和安全性。在本项目中，肺部感染是最常见的感染类型，占比62.5%。药敏分析结果显示，鲍曼不动杆菌、肺炎克雷伯菌、大肠埃希菌和金黄色葡萄球菌对依拉环素的敏感率分别达95.5%、92.5%、72.1%和92.5%（ChinaCAST折点），充分验证了依拉环素的强大抗菌活性。相比之下，鲍曼不动杆菌、肺炎克雷伯菌、大肠埃希菌和金黄色葡萄球菌对替加环素的敏感率分别达77.2%、75.6%、88.0%和81.8%（FDA折点）。

在病原分布方面，以碳青霉烯耐药鲍曼不动杆菌、肺炎克雷伯菌为主；患者情况表现为基础疾病多，病情严重程度高。

治疗结束时，依拉环素整体治疗有效率为90.1%，ICU、血液科、移植科、感染科的临床治疗有效率分别为88.7%、88.8%、94.4%及95.5%。从病原体分析来看，依拉环素对鲍曼不动杆菌、肺炎克雷伯菌的治疗有效率分别为91.4%和91.2%。治疗结束时，微生物学总有效率为90.7%；从第30天感染转归来看，总临床有效率达85.9%，其中鲍曼不动杆菌临床有效率达86.0%，肺炎克雷伯菌临床有效率达86.7%；患者预后分析显示，患者好转出院率达83.0%。

对于依拉环素非敏感的鲍曼不动杆菌和肺炎克雷伯菌感染患者，其微生物学总有效率仍可达96.2%和90.5%。

在安全性方面，依拉环素显示出良好的耐受性。3369例患者中91例上报不良反应，总体不良反应发生率仅为2.7%。

使用依拉环素时，94.6%的医生选择了标准剂量治疗，即1mg/kg q12h，4.3%的医生选择50mg q12h治疗，治疗疗程有所差异；标准剂量治疗的平均治疗疗程为9.0天。

国家药敏专委会（ChinaCAST）已明确依拉环素的敏感性临床折点。项目结果提示，依拉环素在真实世界中的抗菌活性较强，即使按当前折点，仍有非敏感细菌感染患者对依拉环素治疗有效，值得进一步研究。

**Reference:**

1. Connors KP, Housman ST, Pope JS et al. Phase I, open-label, safety and pharmacokinetic study to assess bronchopulmonary disposition of intravenous eravacycline in healthy men and women. *Antimicrob Agents Chemother* 2014; **58**: 2113-8.

2. Singh R, Falcao N, Sutcliff J et al. Plasma protein binding of eravacycline in mouse, rat, rabbit, cynomolgus monkey, African green monkey and human using microdialysis. *53rd Intersci Conf Antimicrob Agents Chemother*, 2013.

3. Kiem S, Schentag JJ. Interpretation of antibiotic concentration ratios measured in epithelial lining fluid. *Antimicrob Agents Chemother* 2008; **52**: 24-36.

4. Lindbom L, Pihlgren P, Jonsson EN. PsN-Toolkit--a collection of computer intensive statistical methods for non-linear mixed effect modeling using NONMEM. *Comput Methods Programs Biomed* 2005; **79**: 241-57.

5. Beal SL. Ways to fit a PK model with some data below the quantification limit. *J Pharmacokinet Pharmacodyn* 2001; **28**: 481-504.

6. Ahn JE, Karlsson MO, Dunne A et al. Likelihood based approaches to handling data below the quantification limit using NONMEM VI. *J Pharmacokinet Pharmacodyn* 2008; **35**: 401-21.

7. Byon W, Smith MK, Chan P et al. Establishing best practices and guidance in population modeling: an experience with an internal population pharmacokinetic analysis guidance. *CPT Pharmacometrics Syst Pharmacol* 2013; **2**: e51.

8. Keizer RJ, Karlsson MO, Hooker A. Modeling and Simulation Workbench for NONMEM: Tutorial on Pirana, PsN, and Xpose. *CPT Pharmacometrics Syst Pharmacol* 2013; **2**: e50.

9. Jonsson EN, Karlsson MO. Xpose--an S-PLUS based population pharmacokinetic/pharmacodynamic model building aid for NONMEM. *Comput Methods Programs Biomed* 1999; **58**: 51-64.

10. Lindbom L, Ribbing J, Jonsson EN. Perl-speaks-NONMEM (PsN)--a Perl module for NONMEM related programming. *Comput Methods Programs Biomed* 2004; **75**: 85-94.
